# Individual- and group-level sex ratios under local mate competition: consequences of infanticide and reproductive dominance

**DOI:** 10.1093/evlett/qrac005

**Published:** 2023-02-09

**Authors:** Jussi Lehtonen, Serena Malabusini, Xiaomeng Guo, Ian C W Hardy

**Affiliations:** Department of Biological and Environmental Science, University of Jyväskylä, Jyväskylä, Finland; Department of Food, Environmental and Nutritional Sciences (DeFENS), University of Milan, Milan, Italy; College of Plant Protection, Nanjing Agricultural University, Nanjing, Jiangsu province, PR China; Department of Agricultural Sciences, University of Helsinki, Helsinki, Finland

**Keywords:** group reproduction, extreme sex ratio skew, dominance, infanticide, sex ratio evolution, local mate competition, *Sclerodermus*

## Abstract

Extremely female-biased sex ratios of parasitoid wasps in multiple-foundress groups challenges evolutionary theory which predicts diminishing bias as foundress numbers increase. Recent theory based on foundress cooperation has achieved qualitative rather than quantitative success in explaining bias among parasitoids in the genus *Sclerodermus*. Here, we develop an explanation, expanding the theory of local mate competition, based on the observation that male production seems dominated by some foundresses within groups. Two sex ratio effects arise from such reproductive dominance: an immediate effect via suppression of male production, and a long-term evolutionary response to reproductive skew. We analyze the outcome of these effects at the individual and group level, the latter being more readily observable. Three model scenarios are analyzed: (1) random killing of developing sons in a group by all foundresses, without reproductive skew, (2) the development of reproductive dominance by some foundresses after sex allocation decisions by all foundresses have been implemented, and (3) reproductive dominance within foundress groups before sex allocation decisions are implemented. The 3 scenarios have subtly different implications for sex ratio evolution, with Models 2 and 3 being novel additions to theory, showing how reproductive dominance can alter the outcome of sex ratio evolution. All models match observations in their outcomes better than other recently proposed theory, but Models 2 and 3 are closest to observations in their underlying assumptions. Further, Model 2 shows that differential offspring mortality after parental investment can influence the primary sex ratio even when random with respect to parental and offspring characters, but targeted at entire clutches. The novel models are solved for both diploid and haplodiploid genetic systems, and confirmed with simulations. Overall, these models provide a feasible explanation for the extremely female-biased sex ratios produced by multi-foundress groups and expand the scope of local mate competition theory to consider reproductive dominance.

## Introduction

Sex ratio theory, beginning with the work of Düsing (1884) and Fisher (1930) (see Gardner, 2023, for an overview of the early history of the field) and later extended by Hamilton (1967) and many others (West, 2009), has been a highly successful area of evolutionary biology. The local mate competition (LMC) model of Hamilton (1967) predicts that when reproduction takes place in groups, such that sons and daughters of a limited number (*n*) of foundresses per group mate, and the daughters then disperse, the evolutionarily stable sex ratio under a diploid or haploid genetic system is $\frac{n-1}{2n}$. As *n* increases, the sex ratio should thus rapidly approach 0.5, which is the sex ratio predicted by earlier models that assume mating occurs throughout the population (panmixia) rather than exclusively within natal groups (Düsing, 1884; Fisher, 1930). It is generally accepted that under panmixia, these sex ratio predictions are not altered by differential mortality of daughters and sons after the period of parental investment (Leigh, 1970; Pirrie & Ashby, 2021) and this very general result has been said to hold provided mortality is random with respect to parental character (West, 2009 p.19). Under LMC, male-biased mortality can influence the sex ratio if it results in mate-limitation for females (Lehtonen & Schwanz, 2018; Nagelkerke & Hardy, 1994).

There is much empirical evidence to support current sex ratio theory (Hardy, 2002; West, 2009) but nonetheless it has been challenged by empirical observations of very female-biased sex ratios when the number of foundresses in a group is large. One such sex ratio puzzle was recently solved by the finding that the consistently female biased sex ratios produced by species in the parasitoid genus *Melittobia* (Hymenoptera: Eulophidae) was due to foundresses in laboratory experiments behaving as if they had not dispersed from their natal areas and attuning their sex allocation toward the extreme female bias that is selected when reproducing with close-relative co-foundresses (Abe et al., 2021; Gardner & Hardy, 2020).

A remaining puzzle is the extent of the sex ratio bias produced by species in the cooperatively brooding (“quasi-social”) parasitoid genus *Sclerodermus* (Hymenoptera [sub-clade Aculeata]: Bethylidae): sex ratios at offspring maturity are typically around 0.1 (10% offspring are males) and appear little influenced by the number of co-reproducing foundresses (e.g., Abdi et al., 2020b, 2021; Guo et al., 2022; Kapranas et al., 2016; Malabusini et al., 2022; Tang et al., 2014; Wang et al., 2016; Wei et al., 2017; Yang et al., 2018) or by the relatedness between foundresses (Abdi et al., 2020a, b; Guo et al., 2022). A proposed explanation (Tang et al., 2014) is that mutually beneficial actions of co-foundress females during host attack and subsequent brood tending could select for the female bias via local resource enhancement (LRE: Taylor, 1981) but the size of the effect predicted by a recent formal model (Iritani et al., 2021) is insufficient to explain the observed bias.

Here, we derive alternative theoretical explanations for extremely female-biased *Sclerodermus* sex ratios, expanding the theory of local mate competition to explore the possible effects of infanticide and reproductive dominance. We summarize the salient aspects of the *Sclerodermus* life-history before describing the models. While these models are directly aimed at understanding the reproductive biology of *Sclerodermus*, they also contribute to sex ratio theory a consideration of reproductive interference between foundresses, where reproduction by a given foundress is altered by the direct actions of other reproductively active foundresses. In the Discussion, we also briefly highlight connections to sex ratio patterns, and their explanations, among some mammals that experience resource competition and exhibit reproductive dominance. Our models also show that even under panmixia, male-biased mortality after parental investment can in principle alter primary sex ratio predictions if mortality is clutch-specific, or reproductive potential of sons is otherwise skewed among foundresses.

### Reproductive biology of Sclerodermus

Species in the genus *Sclerodermus* are unusual among parasitoid hymenopterans in that multiple adult females (foundresses) attack the same host, suppressing it via stinging and paralysis. Eggs are laid externally onto the host and the foundresses remain together with the communal brood for extensive periods (several weeks) while the eggs hatch, the larvae feed on the host and then pupate around the host’s remains. Foundresses actively tend the brood, assisting the commencement of larval feeding by puncturing the host’s integument, arranging larvae on the host, replacing dislodged larvae, removing dead larvae, and assisting offspring with moving away from the host to pupate and then with exiting their pupal cocoons (Gao et al., 2016; Hu et al., 2012; Skvarla, 2018; Tang et al., 2014; Wu et al., 2013; Yang et al., 2012).

While foundresses cooperate in aspects of reproduction, and can on average benefit from reproducing together rather than as single foundresses (Tang et al., 2014), there is evidence for exploitative interactions between foundresses. Foundresses are reported to attune cooperation in host attack to the magnitude of the risks they take and their relatedness to co-foundresses (Abdi et al., 2020a, 2020b; Liu et al., 2021; Mesterton-Gibbons & Hardy, 2021). Recent experimental observations show that, as foundress groups form on a suppressed host, incoming foundresses are most often accepted by foundresses that are already present but also that potential co-foundresses are sometimes aggressively repelled and even killed (Guo et al., 2023). Further evidence indicates that females often contribute similar numbers of offspring to communal broods (low reproductive skew overall) but also that the production of male offspring can be dominated by larger or earlier reproducing foundresses (high skew in male production) (Guo et al., 2022). Several studies have found that the mean number of adult males produced per foundress in each brood is less than one (Malabusini et al., 2022; Tang et al., 2014), indicating that not producing any sons might be a common occurrence for foundresses. These results suggest that some foundresses may be intrinsically dominant or become dominant during the brood production process, and that such dominance is manifest as a manipulation of sex ratio. One potential mechanism of sex ratio adjustment is sexually differential infanticide during the brood tending period. While there is no direct evidence for this, infanticide is within the behavioral repertoire of *Sclerodermus* foundresses, with foundresses sometimes eating eggs, larvae, and pupae from broods they have been tending (Chen, 2016; He et al., 2007). Such infanticide has been observed in the context of a maternal response to damaged offspring or failing broods on decaying, or otherwise unsuitable, hosts (Hu et al., 2012; Jucker et al., 2020; Lupi et al., 2017; Wu et al., 2008; Zhou et al., 1997; S.M. personal observation), in the context of reducing the size of broods which are apparently too large to be supported by a given host (Zhou et al., 1997) and also in the context of an intruding foundresses eating substantial numbers of eggs and larvae belonging to broods from which the mothers had been experimentally removed (X.G., personal observation) (see also Chen, 2016). The models we present here formalize suggestions presented in Guo et al. (2022) and Malabusini et al. (2022) that, under a locally mating population structure, inter-foundress dominance and/or infanticide might explain the brood sex ratios observed in *Sclerodermus* species when offspring mature.

## Models and results

We model sex ratio evolution under local mate competition with a subset of foundresses dominating male production, under a variety of assumptions. The scenarios considered in Models 2 and 3 are modeled analytically under haploid, diploid, and haplodiploid genetic systems and analytical results are confirmed using simulations (diploid/haploid derivations are presented in the main text, while haplodiploid derivations and simulations are presented in the Supplementary Information; central analytical results are presented in Table 2 in the main text). Dominance can take the form of some females adjusting the sexual composition of the offspring produced by others, via sexually differential infanticide (post-sex allocation dominance), or it can take the form of some females controlling the sex allocation decisions of others (pre-sex allocation dominance). However, we begin with a simpler model where there is no reproductive dominance per se and infanticide of immature males within communally produced broods occurs at random (Model 1). We then assume that there is inter-foundress dominance but also that foundresses do not “know” their dominance status when they make their sex allocation decisions, or that a plastic response is not possible for other reasons. All foundresses thus make similar sex allocation decisions (primary sex ratio) and the sons of some (subsequently subordinate) foundresses are then eliminated by other (subsequently dominant) foundresses (Model 2), affecting the secondary sex ratio. Finally, we consider a scenario in which all foundresses know their status prior to reproduction and can evolve flexible sex allocation strategies dependent on their status as a dominant or as a subordinate (Model 3).

**Table 2.** Expressions for individual- and group-level sex ratios for Models 2 and 3 under alternative genetic systems.

		Post-sex allocation dominance (Model 2)		Pre-sex allocation dominance (Model 3)	
		I	II	III	IV
		Primary sex ratio of dominants and subordinates	Group-level (secondary) sex ratio with no mortality of sons of dominants	Primary sex ratio of dominants (subordinate sex ratio=0)	Group-level (secondary) sex ratio with no mortality of sons of dominants
A	Diplo-diploid (or haploid)	$\frac{(d-1)n}{d-n+2dn}$	$\frac{\left(d-1\right)}{d+n}$	$min\left(1,\;\frac{(d-1)n}{2d^{2}}\right)$	$min\left(\frac{d}{n},\;\frac{\left(d-1\right)}{2d}\right)$
B	Haplo-diploid with arbitrary level of sib-mating (*k*)	$\frac{(d-1)(2-k)n}{dn\left(4-k)+(2-k\right)(d-n)}$	$\frac{\left(d-1)(2-k\right)}{d\left(2-k\right)+2n}$	*	*
C	Haplo-diploid with constant group size *n* (*i.e.k*=1/*n*)	$\frac{(d-1)(2n-1)n}{d\left(4n^{2}+n-1\right)+n-2n^{2}}$	$\frac{\left(d-1)(2n-1\right)}{d\left(2n-1\right)+2n^{2}}$	$min\left(1,\frac{a-\sqrt{b}}{c}\right)$ where $a=n-3dn+d^{2}\left(-1+6n\right)$ $b=n^{2}-6dn^{2}+2d^{3}n(-7+2n)+d^{4}{\left(1+2n\right)}^{2}+d^{2}n(2+13n)$ $c=2d^{2}(-1+4d)$	$min\left(\frac{d}{n},\frac{d}{n}\left[\frac{a-\sqrt{b}}{c}\right]\right)$ where $a=n-3dn+d^{2}\left(-1+6n\right)$ $b=n^{2}-6dn^{2}+2d^{3}n(-7+2n)+d^{4}{\left(1+2n\right)}^{2}+d^{2}n(2+13n)$ $c=2d^{2}(-1+4d)$

*Presenting *k* (the level of sib-mating) as a free parameter in these combinations is not internally consistent, because *k* depends on the sex ratio itself.

The letters A to C and the numerals I to IV correspond to plots presented in Figures 2 to 5.

The method used in the initial analytical models is evolutionary game theory for a continuous trait (Maynard Smith, 1982) (in some respects very similar to adaptive dynamics (see Lehtonen, 2018), which is perhaps the most common contemporary method for developing and solving sex ratio models (West, 2009)). The equations estimate selection on mutants that deviate from the prevalent resident sex ratio strategy by a small amount. In each scenario, we start by deriving an expression for the fitness of a rare mutant with sex ratio x that appears in a population otherwise expressing sex ratio $\hat{x}$. In the haplodiploid models, we also incorporate aspects of kin selection models (Taylor & Frank, 1996) to account for asymmetric relatedness and reproductive values of sons and daughters (see Supplementary Information). We provide predictions for both individual-level and group-level sex ratios. Notation for models and definitions of sex ratio terminology are presented in Table 1. In the Supplementary Information, we also present an alternative inclusive fitness analysis (following Denver & Taylor, 1995; Frank, 1998; Taylor, 1993).

**Table 1.** Terminology and notation.

Term	Definition
Sex ratio	Proportion of offspring that are male
Primary sex ratio	Sex ratio at the time of sex allocation, i.e., at oviposition
Secondary sex ratio	Sex ratio at the time of brood maturity, i.e., at mating
Individual sex ratio	Sex ratio of offspring produced by an individual foundress within a group of *n* foundresses communally reproducing on a patch
Group sex ratio	Overall sex ratio of a brood of offspring communally produced by *n* foundresses on a patch
x	Sex ratio of a rare mutant
$\hat{x}$	“Resident” (non-mutant) sex ratio
$x^{*}$	Evolutionarily stable sex ratio (ESS)
q	Proportion of surviving sons after infanticide in Model 1, or the proportion of sons of dominants surviving after infanticide in Models 2 and 3
n	Total number of foundresses contributing to the communal brood (dominants + subordinates)
d	Number of dominant foundresses contributing to the communal brood. (Only values of *d *> 1 are considered here: *d *= 1 leads to the biologically unrealistic result of no male production, analogous to the prediction for *n *= 1 in the original LMC model Hamilton, 1967, our Equation 2, and additional assumptions would be needed to model the outcome in that scenario).

### Model 1: No reproductive dominance and random infanticide of males

We first model the effects of random killing of immature males when no foundresses are reproductively dominant over others. We do not claim Model 1 to be a novel result in itself but it forms a useful point of departure and comparison for Models 2 and 3, which are novel. Assume a fraction (1-*q*) of the very large number of male eggs in a brood is randomly killed, and a fraction *q* survives. Such evenly divided risk does not require completely indiscriminate killing: if the probability of an encounter and subsequent infanticide between all foundress-son pairs is equal, with the exception that every foundress avoids killing their own sons, the aggregate risk is still divided evenly over sons in a brood. The fitness of a focal foundress under these assumptions is:


$$\begin{array}{l} \begin{array}{ll} w\; & =\left(1-x\right)+qx/\left(qx+\left(n-1\right)q\hat{x}\right)\left[\left(1-x\right)+\left(n-1\right)\left(1-\hat{x}\right)\right]\\ =\left(1-x\right)+x/\left(x+\left(n-1\right)\hat{x}\right)\left[\left(1-x\right)+\left(n-1\right)\left(1-\hat{x}\right)\right] \end{array} \end{array}$$ (1)

Here, the male egg survival terms q cancel out in the term representing mating competition between sons, and the model simplifies to a standard LMC model. Note that Equation (1) depends on the assumption that all females will have mating opportunities with surviving males on maturity (see e.g., Lehtonen & Schwanz, 2018; Nagelkerke & Hardy, 1994, for models that account for the risks of all immature males dying). Here, as well as in Models 2 and 3, the direction of selection is found by differentiating fitness for the mutant value of *x*, i.e., by computing ${\frac{dw}{dx}|}_{x=\hat{x}}$ (Lehtonen, 2018; Maynard Smith, 1982). The candidate for the evolutionarily stable primary sex ratio is found by setting the derivative to zero and solving for *x*, i.e., finding the value of *x* where selection vanishes (Lehtonen, 2018; Maynard Smith, 1982). The solution is


$$\begin{array}{l} x^{*}=\frac{n-1}{2n} \end{array}$$ (2)

where the evolutionarily stable strategy has been indicated with an asterisk. This is the well-known local mate competition result (Hamilton, 1967), showing that mortality of sons in Model 1 has no effect on individual-level evolutionarily stable sex allocation (the primary sex ratio) if, as here assumed, surviving daughters always have mating opportunities.

Despite no evolutionary response on individual-level sex ratio, the observed post-mortality group-level sex ratio (secondary sex ratio) is altered by male mortality:


$$\begin{array}{l} x_{group}^{*}=\frac{nq\left(\frac{n-1}{2n}\right)}{nq\left(\frac{n-1}{2n}\right)+n\left(1-\frac{n-1}{2n}\right)}=\frac{q(n-1)}{q\left(n-1\right)+(n+1)} \end{array}$$ (3)

**Figure 1.**
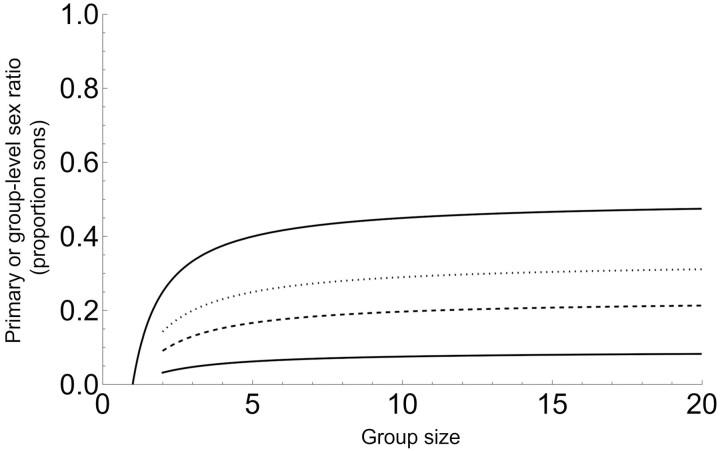
Primary- and group-level (secondary) sex ratio when the killing of immature males is random (Model 1). The top curve is the original LMC result (Hamilton, 1967), our Equation 2, which in this case also corresponds to individual-level primary sex ratio, or to group-level sex ratio when *q* = 1. The lower solid, dashed, and dotted curves are group-level sex ratios (Equation 3) with increasing survival of sons; *q* = 0.1, *q* = 0.3, and *q* = 0.5, respectively.

Figure 1 illustrates that, under the assumptions of Model 1, group sex ratios can be considerably lower than under standard LMC.

### Model 2: Post-sex allocation dominance

We now model a scenario where there are subordinate and dominant foundresses and subordinates make the same sex allocation decisions as dominants. This could happen, for example, if foundresses are unable to predict their future status as a dominant or as a subordinate and make their sex allocation decisions before a dominance hierarchy arises. Once dominance develops, dominant foundresses may adjust the sex ratios produced by subordinates by killing their male offspring before they mature. We initially assume that all sons of subordinates are killed, that dominants may have some (but not all) of their sons randomly killed, and that no daughters (of either dominants or subordinates) are killed. We adopt these assumptions on the basis that it does not pay off to kill the daughters of other foundresses because every daughter killed is a loss of a potential mating partner for a given foundress’s sons (here we assume there is no fitness advantage to offspring associated with developing in smaller broods with greater per capita access to limited nutritional resources) but that it is advantageous for a foundress to reduce competition for mates among her sons and so she does this whenever she is able, i.e., when dominant, by killing the immature male offspring of subordinates. We provide mathematical justification for this intuitive argument in the Supplementary Information, where we show that infanticide of sons can be adaptive provided there is some ability to avoid killing one’s own sons, even if inaccurate. Individuals have no opportunity to adjust sex allocation according to their (future) dominance status, and a dominant female is assumed to be no more likely to produce dominant offspring than a subordinate one (i.e., dominance is not heritable). The fitness of a mutant foundress is now


$$\begin{array}{l} \begin{array}{ll} w= & \;\left(1-x\right)+\left(\frac{d}{n}\right)\left\{qx/\left(qx+\left(d-1\right)q\hat{x}\right)\left[\left(1-x\right)+\left(n-1\right)\left(1-\hat{x}\right)\right]\right\}\\ & =\left(1-x\right)+\left(\frac{d}{n}\right)\left\{x/\left(x+\left(d-1\right)\hat{x}\right)\left[\left(1-x\right)+\left(n-1\right)\left(1-\hat{x}\right)\right]\right\} \end{array} \end{array}$$ (4)

Here we have divided the fitness of the foundress into two parts: the first part accounts for fitness via daughters (which is not dependent on dominance status) and the second for fitness via sons if the foundress is one of the *d* dominants in the group of *n* foundresses, which occurs with probability $\left(\frac{d}{n}\right)$. Due to infanticide of the sons of subordinates, maturing sons are exclusively the offspring of dominant foundresses, while all foundresses may produce daughters. All surviving sons are assumed to compete equally with each other for matings with all daughters produced by all foundresses. Note that analogous to Model 1, the survival probability *q* of the sons of dominants again cancels out in the expression. If each surviving male is subsequently equally likely to gain a given mating (“raffle principle”) then the average fraction of all matings gained by sons of a mutant foundress is $x/\left(x+\left(d-1\right)\hat{x}\right)$, the mating partners being the $\left(1-x\right)+\left(n-1\right)\left(1-\hat{x}\right)$ daughters produced by all foundresses contributing to the group. In the above derivation, all sons of subordinates are killed before maturing, while sons of dominants survive in sufficient numbers to fertilize all daughters: sons of dominants can have some risk of being killed, either by dominant or subordinate foundresses or both, and the result is unaltered, as long as this risk is equal for all dominant foundresses. The direction of selection is found with the same method as in Model 1, and the equilibrium solution is


$$\begin{array}{l} x^{*}=\frac{(d-1)n}{d-n+2dn}=\frac{1-1/d}{2-1/d+1/n} \end{array}$$ (5)

**Figure 2.**
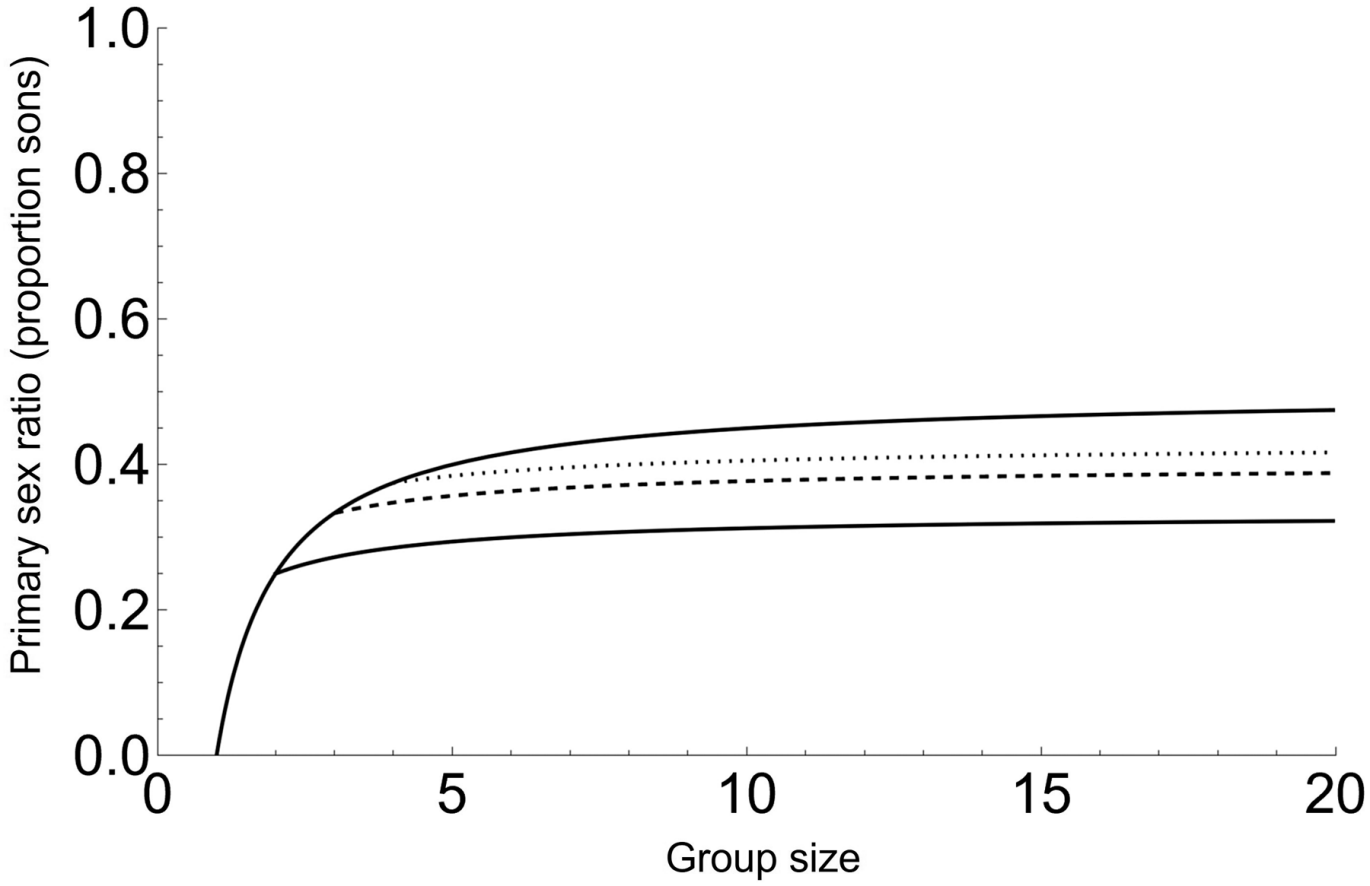
Primary sex ratio of individual foundresses with post-sex allocation dominance (Model 2). The top curve is the original LMC result (Hamilton, 1967), our Equation 2. The lower solid, dashed, and dotted curves correspond to Equation 5 (AI in Table 2) with *d* = 2, *d* = 3, and *d* = 4, respectively.

We examine the components and structure of Equation (5) in relation to earlier models in the Discussion. Figure 2 illustrates that, under the assumptions of Model 2, the sex ratios produced by individual foundresses are lower than under standard LMC when *d* < *n*.

Although we focus mainly on the above result, in the Supplementary Information we show that Equation (5) is in fact a special case of a more general result, where there is among-foundress variance in the competitiveness or survivorship of their male offspring within each patch: if the coefficient of variation within patches of this generalized competitive ability is $c_{v}$, then


$$\begin{array}{l} x^{*}=\frac{1-\left(1+{c_{v}}^{2}\right)/n}{2-{c_{v}}^{2}/n} \end{array}$$ (6)

Thus, when $c_{v}=0$, we find Hamilton’s (1967) LMC result (Equation 2). The dominant/subordinate division described above is a clear and intuitive way in which a positive $c_{v}$ can arise and recovers Equation (5) from Equation (6) while covering the possible range of values of $c_{v}$ (see Discussion and Supplementary Information).

Equation (5) denotes the equilibrium value of the sex ratio trait but that trait is not fully expressed in all individuals: subordinates only produce (mature) female offspring. Given that empirically it is typically more straightforward to observe the overall offspring sex ratio of a group than the component contributions of individual foundresses (e.g., Guo et al., 2022; Tang et al., 2014), it is again useful to calculate the resulting equilibrium average offspring sex ratio, i.e., the overall proportion of males among all offspring in a group at maturity. Note that this can differ from the simple average of the sex ratios of foundresses because in Model 2 not all foundresses have the same number of offspring, due to infanticide.


$$\begin{array}{l} x_{group}^{*}=\frac{dqx^{*}}{dqx^{*}+n\left(1-x^{*}\right)}=\frac{\left(d-1\right)q}{n+1+\left(d-1\right)q} \end{array}$$ (7)

where $x^{*}$ is given by Equation (5). When all dominants survive (*q* = 1) Equation (7) becomes particularly simple, clearly reflecting classic LMC results we obtain:


$$\begin{array}{l} x_{group}^{*}=\frac{\left(d-1\right)}{d+n} \end{array}$$ (8)

**Figure 3.**
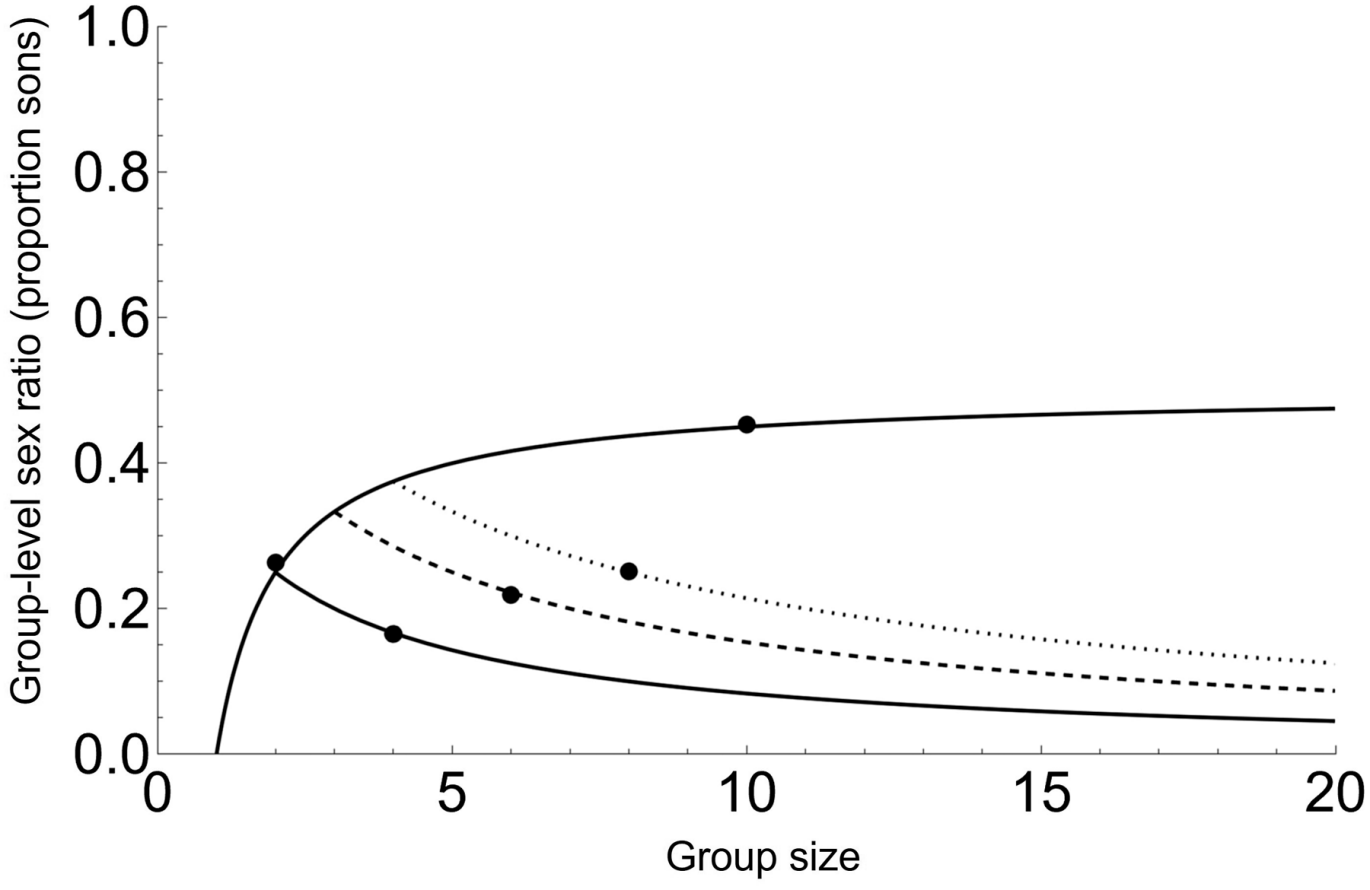
Group-level (secondary) sex ratio with post-sex allocation dominance (Model 2). The top curve is the original LMC result (Hamilton, 1967), our Equation 2. The lower solid, dashed, and dotted curves correspond to Equation 8 (AII in Table 2) with increasing numbers of dominant foundresses within the group; *d* = 2, *d* = 3, and *d* = 4, respectively. The five dots are simulation results, confirming analytical results for a selection of parameter values (from left to right: *n* = 2, *d* = 2; *n* = 4, *d* = 2; *n* = 6, *d* = 3; *n* = 8, *d* = 4; and *n* = 10, *d* = 10).

Equation (8) represents an upper limit to the group sex ratio of Equation (7). Figure 3 illustrates that even when we use the upper limit of Equation (8), predicted group secondary sex ratios can be considerably lower than under standard LMC and, for large numbers of foundresses, are typically around 0.1 to 0.2 (10%–20% of maturing offspring are males).

### Model 3: Pre-sex allocation dominance

In this model, we assume that subordinates “know” they are subordinates and also “know” that dominants will not allow them to produce adult male offspring (but dominance is, again, assumed to be not inherited or correlated to any heritable trait). Under this scenario, intuition suggests that it pays off for subordinates to allocate all their reproductive resources toward daughters. It is much more difficult to intuit the corresponding long-term coevolution of the dominant strategy: we model this both analytically (below) and with simulations (Supplementary Information). Assuming the evolution of a plastic response to dominance status (i.e., different strategies are expressed in dominant and subordinate individuals), the fitnesses for dominants and subordinates, respectively, are


$$\begin{array}{l} \left\{ \begin{array}{l} w_{d}=\left(1-x_{d}\right)+qx_{d}/\left(qx_{d}+\left(d-1\right)q{\hat{x}}_{d}\right)\left[(1-x_{d})+\left(d-1\right)\left(1-{\hat{x}}_{d}\right)+\left(n-d\right)\left(1-x_{s}\right)\right]\;\\ w_{s}=\left(1-x_{s}\right)+0/\left(0+\left(d-1\right)x_{d}\right)\left[(1-x_{s})+(d-1)\left(1-x_{d}\right)+(n-d)(1-{\hat{x}}_{s})\right]=\left(1-x_{s}\right)\; \end{array}\right. \end{array}$$ (9)

where the coefficient *q* cancels out as in Models 1 and 2, i.e., random mortality of sons of dominants does not alter the outcome. The dominant and subordinate sex ratios and fitnesses are now indicated with subscripts *d* and *s*. $w_{s}$ is clearly a decreasing function of $x_{s}$ (with $\frac{dw_{s}}{dx_{s}}=-1$), so, as expected, the subordinate (primary) sex ratio evolves to its minimum value of 0, i.e., no male production (at oviposition). The equilibrium for dominants is then found by substituting $x_{s}=0$ into Equation (9) and subsequently finding the value of $x_{d}$ for which ${\frac{dw_{d}}{dx_{d}}|}_{x_{d}={\hat{x}}_{d}}=0$. Thus, we find the primary sex ratios


$$\begin{array}{l} \left\{ \begin{array}{l} {x_{d}}^{*}=min\left(1,\;\;\;\frac{\left(d-1\right)n}{2d^{2}}\right)\\ {x_{s}}^{*}=0\; \end{array}\right. \end{array}$$ (10)

where we have taken into account (using min) that with some combinations of *n* and *d* the expression $\frac{(d-1)n}{2d^{2}}$ could take on values above 1 which is biologically not possible. The correct interpretation in such cases is that selection to increase (or maintain) investment in sons is positive over the entire range of x, and xwill be driven to its maximum value 1 under some circumstances

**Figure 4.**
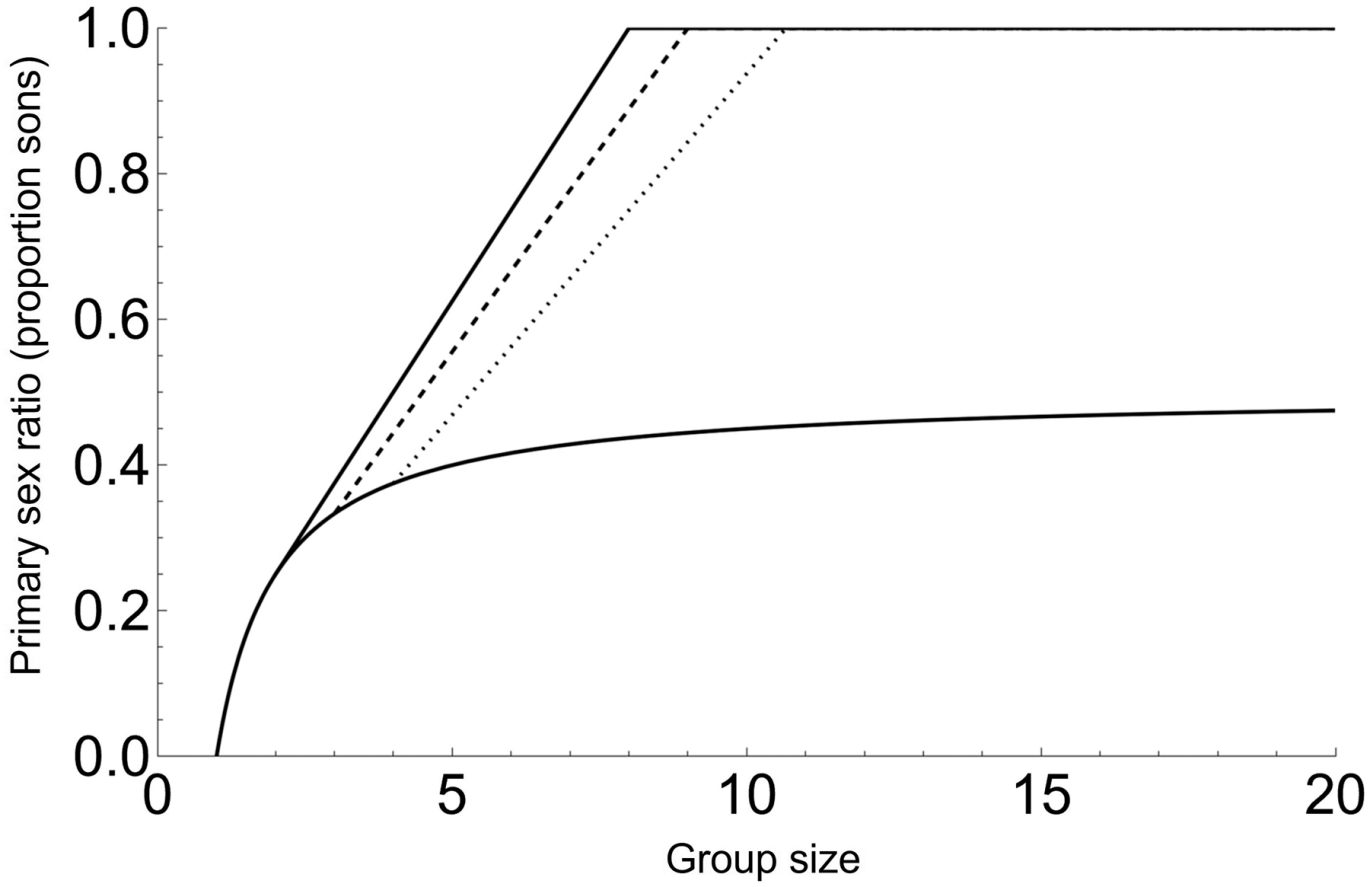
Primary sex ratio of dominants with pre-sex allocation dominance (Model 3). The lower curve is the original LMC result (Hamilton, 1967), our Equation 2. The upper solid, dashed, and dotted lines correspond to Equation 10 (AIII in Table 2) with increasing numbers of dominants; *d* = 2, *d* = 3, and *d* = 4, respectively. Under this model, the primary sex ratio of subordinates is 0 (not illustrated).

Figure 4 illustrates that, under the assumptions of Model 3, the sex ratios produced by dominant foundresses are considerably higher than under standard LMC, and that such foundresses should produce exclusively male offspring when a small number of dominants reproduce along with a large number of subordinates. Again, we provide a conceptual analysis of these equations in the Discussion.

As before, it is useful, for comparison to empirical results, to calculate an average of Equation (10) across all offspring (i.e., those of both dominants and subordinates) in a group:


$$\begin{array}{l} x_{group}^{*}=min\left(\frac{dq}{n+d(q-1)},\;\;\;\frac{(d-1)q}{d+1+(d-1)q}\right) \end{array}$$ (11)

As was the case with Model 2, when all offspring of dominants survive (*q* = 1), Equation (11) becomes particularly simple, clearly reflecting classic LMC results:


$$\begin{array}{l} x_{group}^{*}=min\left(\frac{d}{n},\;\;\;\frac{\left(d-1\right)}{2d}\right) \end{array}$$ (12)

**Figure 5.**
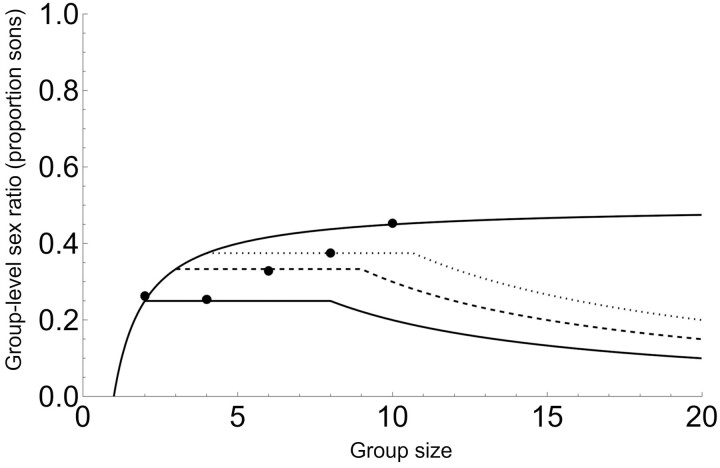
Group-level (secondary) sex ratio with pre-sex allocation dominance (Model 3). The top curve is the original LMC result (Hamilton, 1967), our Equation 2. The lower solid, dashed, and dotted curves correspond to Equation 12 (AIV in Table 2) with increasing numbers of dominants; *d* = 2, *d* = 3, and *d* = 4, respectively. The five dots are simulation results, confirming analytical results for a selection of parameter values (from left to right: *n* = 2, *d* = 2; *n* = 4, *d* = 2; *n* = 6, *d* = 3; *n* = 8, *d* = 4; and *n* = 10, *d* = 10).

Equation (12) represents an upper limit to the group sex ratio under the assumptions of Model 3, while random mortality of sons of dominants (*q* < 1) can only decrease the group sex ratio even further. Figure 5 illustrates that under the assumptions of Model 3 and *q* = 1, predicted group sex secondary ratios can be considerably lower than under standard LMC and, for large numbers of foundresses, are typically around 0.1–0.3 (10%–30% of maturing offspring are males) and are most biased when few dominants are present among large numbers of subordinate foundresses.

### Genetic system

In Table 2 and in the Supplementary Information, we present further modifications to our main models. The sex ratio solutions presented so far implicitly assume diploid or otherwise symmetric inheritance (e.g., a hypothetical haploid genetic system). If inheritance is haplo-diploid, it is known from previous theory that the ESS of LMC models can be slightly altered (Frank, 1985; Hamilton, 1979; Taylor & Bulmer, 1980; West, 2009), thus making it important to investigate the magnitude of this effect in our model. Haplodiploidy has this effect because consanguinity of a parent and its diploid offspring is altered by inbreeding (West, 2009) but the same does not apply to a parent and its haploid offspring: an inbred diploid offspring has an elevated probability of inheriting two identical copies of an allele from its two parents, whereas a haploid offspring only ever inherits one copy from its mother, irrespective of the level of inbreeding. The expected number of gene copies carried by diploid offspring therefore increases with inbreeding, whereas it remains the same in haploid offspring. The outcome is that if males and females are of the same ploidy, their relative genetic relatedness to the mother remains unchanged under inbreeding but in a haplo-diploid system (males are haploid and females are diploid) this assumption is violated. This causes a slight increase in allocation to female offspring in classic LMC models which correspond to Model 1 (West, 2009) as well as the novel Models 2 and 3 presented here (Table 2 and Supplementary Information). We do not present figures for the haplodiploid model in the main text for two reasons: (a) the level of sib-mating over evolutionary history is not known for *Sclerodermus* and (b) the difference compared to the diploid model is relatively small even when inbreeding is high, and likely smaller than error in empirical measurement (Supplementary Information). The substantially more transparent diploid model is thus sufficient to present the key results. The diploid model, with its simpler solutions, provides a reasonable upper limit with equations that remain relatively intuitive, with clear parallels to the classic local mate competition result (Equation 2).

## Discussion

Our results have both theoretical and empirical relevance, which we discuss in turn.

### Theoretical aspects

From a theoretical point of view, our results expand the understanding of sex ratio theory, with strong ties to earlier models of local mate competition. First, if mortality of sons within a maturing group is random (e.g., son-foundress pairs encounter each other randomly, and a foundress kills sons of other foundresses on encounter), Hamilton’s result (Hamilton, 1967; West, 2009) for the unbeatable sex ratio (Equation 2) is replicated exactly, assuming that sufficient sons survive to fertilize all daughters in the patch. The latter is a reasonable assumption when brood sizes are large, as is the case with *Sclerodermus* (e.g., Figure 6 in Abdi et al. [2020a]), and when the number of foundresses is high (which is where the previously unexplained empirical results mainly occur). We do not claim Model 1 to be a novel finding in itself, but rather that it provides results for comparison with the novel Models 2 and 3. However, even in the simple scenario of Model 1, the group-level sex ratio (the most immediately empirically observable quantity) is altered by random male infanticide (Equation 3): if sons are killed while daughters are not, the secondary sex ratio inevitably becomes more female-biased.

The more interesting cases are those where male infanticide induces an evolutionary response at the individual level. This happens if reproduction via sons is skewed such that some foundresses in a patch have more success per son than others. At the most general level, in Model 2, the sex ratio can be shown to depend on the coefficient of variation of this success via sons. One mechanism which can cause such reproductive skew is if some foundresses are dominant, such that they are either able to kill developing sons of subordinates or prevent them from being produced in the first place. We have used the terminology of “dominants” and “subordinates” for clarity and convenience; however, the necessary assumption is skewed reproduction via sons, not of dominance per se. It is also noteworthy that in Model 2 with male infanticide, all newly laid male eggs have identical chances of survival, and every mother making her sex ratio decisions has equal chances of her sons surviving. Mortality is therefore distributed randomly with respect to parental and offspring character, a condition which has been thought to prevent any effect on the ESS sex allocation under panmixia (West, 2009). Under LMC, male-biased mortality is known to change sex allocation predictions if fertilization of all daughters is not guaranteed (Lehtonen & Schwanz, 2018; Nagelkerke & Hardy, 1994). Yet the effect on sex ratio in our Model 2 is present in both patch-structured and panmictic populations (see below), when all daughters are fertilized. The model therefore adds an interesting twist to the generally accepted result that differential mortality after parental investment should not influence sex ratio evolution (Pirrie & Ashby, 2021; West, 2009).

The form $x^{*}=\frac{1-1/d}{2-1/d+1/n}$ for the solution to Model 2 provides conceptual insight into our result and connects it clearly to previous work. Consider the terms 1/d and 1/n. The former relates to the extent of local mate competition (i.e., the sons of *d* compete), while the latter relates to the extent of sib-mating (i.e., at conception, a randomly picked daughter has a probability 1/*n* of mating with a brother—see Supplementary Information). This pinpoints one central feature of the model: local mate competition and sib-mating are decoupled. For example, in the derivation and notation of Maynard Smith (1978, p.160–161) and Taylor (1993) we would have $\frac{1}{d}=\frac{1}{n}=k$: a single parameter for sib-mating, in which case two terms in the denominator cancel out. Alternatively, in models where sib-mating is avoided entirely (either due to female dispersal prior to mating (Werren, 1983; West, 2009 p.155), due to explicit sib-mating avoidance (West, 2009 p.155), or if selfing is avoided in hermaphrodites (Charnov, 1980, an example where the ‘offspring’ (gametes) are haploid), in terms of Equation (5) we would have 1/*n* = 0. In either case, part of the causal structure of (5) is hidden. It is this decoupling of the two terms that also makes it possible for male-biased mortality to influence sex ratio evolution even under panmixia, where we might (at least in principle) have the sib-mating term approach zero, while the competition term can remain positive, which obtains a sex ratio of $\frac{1-1/d}{2-1/d}=\frac{d-1}{2d-1}$. Therefore, even in a panmictic population, sexually differential mortality after parental investment can influence the sex ratio, provided it is not evenly distributed over clutches. Such a result under panmixia is, however, likely empirically undetectable: the deviation from the Fisherian value of ½ would only be notable with a small value of *d*, which seems less likely in a panmictic population than in a patch-structured scenario.

An alternative, equivalent, interpretation is that 1/n is the expected relatedness (relative to her own sons) of a foundress to a randomly picked son in the patch at conception, prior to infanticide or other dominance interactions, while 1/d can be interpreted as the expected relatedness of a dominant foundress to a randomly picked son competing for matings in the patch (relative to her own sons). We show this using an inclusive fitness derivation in the Supplementary Information. The interpretations ultimately measure the same thing: local mate competition is strong when sons commonly compete with their brothers, and sib-mating is prevalent when a foundress (and hence also her daughter) is closely related to a randomly picked son in the patch.

Equation (6) takes a different and more general view on Model 2: it shows that the sex ratio in Model 2 depends only on group size *n* and the coefficient of variation ($c_{v})$ among foundresses in reproductive potential via sons. Because $c_{v}$ is dimensionless, the simple expression $x^{*}=\frac{1-\left(1+{c_{v}}^{2}\right)/n}{2-{c_{v}}^{2}/n}$ shows that only the relative magnitudes in this distribution of reproductive potential matter for the sex ratio, not their absolute magnitudes. In other words, if one distribution yields a given sex ratio, we can multiply that distribution by any positive coefficient and obtain the same result (again, assuming that all daughters are fertilized). Similarly, many distributions of different shapes will yield the same sex ratio provided that their coefficient of variation is identical. This also implies that our basic version of Model 2 with *d* dominants in a group of *n* explores the whole range of possibilities: Katsnelson and Kotz (1957) showed that the coefficient of variation of *n* non-negative numbers can vary in the range $\left[0,\sqrt{n-1}\right]$. With the setup of *d* dominants out of *n*, we obtain $c_{v}=0$ when *d* = *n*, and $c_{v}=\sqrt{n-1}$ when *d* = 1 (see Supplementary Information). The simple setup of *d* dominants is therefore at the same time biologically intuitive and sufficiently general to cover the whole range of sex ratios that the general equation (Equation 6) could yield.

Moving on to Model 3, Equation (10) has a very different interpretation. Intuitively, it is clear that subordinates do not benefit from allocation into males, and selection acts against this when allowed to evolve independently in subordinates, hence ${x_{s}}^{*}=0$. This in turn implies that there are (*n*-*d*) subordinate females producing only daughters, which only the sons of *d* dominants compete for. Without these additional daughters, the dominants would effectively evolve in groups of *d* foundresses and evolve a standard LMC sex ratio of $\frac{\left(d-1\right)}{2d}$, but the “free” extra daughters increase the *per capita* reproductive value of sons. We can rewrite the sex ratio of dominants as ${x_{d}}^{*}=\frac{(d-1)n}{2d^{2}}=\frac{\left(d-1\right)}{2d}(1+\frac{n-d}{d})$. In other words, the additional allocation into sons by dominant foundresses is proportional to the relative number of additional daughters of subordinates that are available to the sons of dominants $(\frac{n-d}{d}).$ The seemingly subtle difference of prior self-knowledge of dominant or subordinate status therefore has a major effect on the resulting sex allocation and, in both cases, the results differ from classic LMC results at the individual and group levels. Our findings are therefore in line with previous models where information availability has been found to influence evolutionary outcomes (Abe et al., 2003; Pen & Taylor, 2005; Stubblefield & Seger, 1990). Note, however, that even in Model 3, where individuals “know” their own status, they have no information about the strategies of other individuals (compare to, e.g., Abe et al., 2003, a two-foundress model where a foundress can recognize another’s sex ratio), and the models do not involve any form of “negotiation” (compare to, e.g., Pen and Taylor, 2005, an eusocial model where workers and queens may be able to recognize each other’s reproductive allocation strategies). It may be of interest in future work to construct models similar to ours where *Sclerodermus* foundresses are able to observe and respond to each other’s sex allocation strategies (in similar vein to Kamimura et al. (2008), a model where foundresses, with no differences in dominance status, can cooperate in sequential bouts of sex allocation and non-cooperative cheating is policed and responded to by the production of less female biased sex ratios; or to Wakano (2005), where females dynamically produce different numbers of males (that may engage in combat with each other) at different times during their reproductive period). The “Stackelberg equilibrium” approach of, e.g., Pen and Taylor’s (2005) Model 2 is another intriguing future avenue for extending our models, and to explicitly consider early and late reproducing individuals and the coevolution of their sex ratio strategies. Although the sex ratio of individual mothers predicted under the conditions considered in Models 2 and 3 differ considerably (Figures 2 and 4), the resulting group sex ratios are broadly similar (Figures 3 and 5); empirical distinction between the predictions of these models may thus require direct assessment of the primary sex ratio (e.g., Khidr et al., 2013).

There is some similarity between our models and the Trivers-Willard hypothesis (Trivers & Willard, 1973), which predicts that a mother in good condition should adjust her sex allocation to produce more males, while a mother in poor condition should produce more females. In that scenario, sons benefit from maternal investment more greatly than do daughters, with the benefits to sons manifest as an advantage when competing against other males for mates (sons of good-condition mothers are thus intrinsically better competitors). In our Model 2 scenarios, all mothers produce the same primary sex ratio and therefore cannot individually adjust their sex ratio according to their condition (dominance status) as they do in the Trivers-Willard hypothesis. In Model 3, however, condition-dependent sex allocation is possible: a dominant mother produces sons and prevents other mothers from so doing, and dominants and subordinates can adjust their sex ratios separately. We may interpret this by viewing the sons of dominants not as intrinsically better competitors than other males but, facing reduced competition at maturity, they have substantially better mating opportunities. Another interpretation (closer to the Trivers-Willard hypothesis) is that the sons of dominants are indeed intrinsically better competitors, by dint of having better survival prospects bestowed upon them “extrinsically” by the actions of their mothers (and other dominant foundresses) toward the sons of subordinates. Frank (1987) showed that under Trivers-Willard type scenarios in which the reproductive returns on investment in sons and daughters differ and the amount of resource available for reproduction vary among mothers, mothers should adjust their sex allocation conditional on the resources available to them, and that the sex ratios of individual mothers and of the population should depend on how resources are distributed across individuals. Our Model 3 analogously predicts that mothers should adjust their sex allocation conditional on whether dominance status is available to them, and that the sex ratios of individual mothers and of the population should depend on how dominance is distributed across individuals.

While the individual-level sex ratio results in Models 2 and 3 are more complicated than the usual LMC expression for sex ratio, they have clear causal interpretations, as explained above. Furthermore, the resulting group-level sex ratios for symmetric (haploid or diploid) genetics are very simple and reminiscent of the LMC expression (compare Equations 2, 8, and 12 in particular). The equivalent results for haplo-diploidy (Table 2) are substantially more complex, but nonetheless important, as many relevant empirical systems (including parasitoid wasps such as *Sclerodermus*) are haplo-diploid. The results for symmetrical genetic systems remain valuable for their transparency and differ relatively little from the haplo-diploid predictions (Supplementary Information). Because of this simplicity and transparency, we have presented the main results with the model for symmetrical genetic systems, which also allows straightforward comparison to the original LMC model (Hamilton, 1967) in terms of the results and the structure of the expressions. The results of Models 1–3 all reflect the standard LMC model (Hamilton, 1967) in a transparent way. In fact, Model 1 is the standard LMC model but with random mortality of males explicitly accounted for. Models 2 and 3 connect to standard LMC in two ways. First, if we set *d* = *n* in Equations 5, 8, 10, or 12, we recover the result $\frac{\left(n-1\right)}{2n}$ in all four cases. This happens because (a) when all foundresses are equally dominant, the biology of the model matches that of LMC, where all foundresses can freely produce daughters and sons and (b) under this scenario, where all foundresses are allowed to reproduce freely and identically, the individual-level and group-level sex ratios must at equilibrium coincide. Second, a more intriguing connection is the structure of the group-level equations (when *q* = 1) in Models 2 and 3, $\frac{\left(d-1\right)}{d+n}$ and $\frac{\left(d-1\right)}{2d}$, which both differ from the simplicity of LMC by only a minor change: in the former, the roles of *d* and *n* become intermixed, and in the latter, *n* is replaced by *d*. Note that selection is modeled as individual-level selection, not group selection, but this individual-level selection model leads to a result that is seemingly simpler at the group level.

### Empirical relevance

Models 1, 2, and 3 all suggest possible explanations for very female-biased sex ratios that have been consistently observed in species in the parasitoid genus *Sclerodermus*. Specifically, they support suggestions that under a locally mating population structure, inter-foundress dominance and/or infanticide might explain the brood sex ratios observed when offspring mature (Guo et al., 2022; Malabusini et al., 2022). Observed *Sclerodermus* sex ratios typically approximate 0.1 and appear little affected by foundress numbers (Abdi et al., 2020a, 2020b, 2021; Guo et al. 2022; Hong et al., 2008; Kapranas et al., 2016; Lupi et al., 2017; Malabusini et al., 2022; Wang et al., 2016; Wei et al., 2017; Yang et al., 2018; Zhao et al., 2020). Our models readily explain group sex ratio biases of around 0.2 and include predictions of around 0.1 in some circumstance. In contrast, the shifts in sex ratio, compared to classical LMC models, predicted by considering the effects of foundresses benefitting mutually from each other’s presence during the brood production process (LRE, Tang et al., 2014), are very small (Iritani et al., 2021). As such, the competitive behaviors of dominance and infanticide seem better explanations for empirical observations than the cooperative behaviors that may lead to local resource enhancement, although we do not consider that the role of mutually beneficial interactions should be abandoned entirely as a candidate to explain *Sclerodermus* sex ratio bias.

While all three models predict group sex ratios that broadly match observations, and Model 1 is the simplest, we do not prefer it on grounds of parsimony. This is because molecular genetic analysis of *Sclerodermus* broods has found that larger or earlier-reproducing foundresses tend to produce all or most of the surviving males within group-produced broods, even when all foundresses produce daughters (Guo et al., 2022). This pattern of individual sex ratios within groups is not captured by Model 1, that considers ovicide without dominance, and therefore Models 2 and 3, that include a form of dominance, fit empirical evidence better (and indeed their construction was stimulated by empirical observations that suggest dominance).

The situation we have considered for groups of foundresses in the parasitoid genus *Sclerodermus*, sex ratios under ovicide and dominance, extends formal sex ratio theory in new ways for group-reproducing organisms. It is nonetheless reminiscent of reproductive conflicts, including conflicts over sex allocation, that have been much studied in other hymenopterans in which there are more-clearly delineated reproductive castes (eusocial wasps, ants, and bees) (e.g., Helanterä & Ratnieks, 2009; Quinones et al., 2020; West, 2009). For example, the sex ratios produced by queens may be altered via ovicide of queen-produced males by workers (Sundström et al., 1996). Workers may also prevent each other from reproducing via aggression or may detect and eat the (inevitably male) eggs laid by other workers (worker policing: Foster & Ratnieks, 2001; Stroeymeyt et al., 2007). Further, with a queen removed, dominance among workers may develop and high-ranking individuals may begin to reproduce (Stroeymeyt et al., 2007). These studies also demonstrate that at least some aculeate hymenopterans have evolved the types of discriminatory abilities and behaviors that are assumed by our models (e.g., the recognition of the sex or parentage of immature offspring, Helanterä & Sundström, 2005; Kamimura et al., 2008; Helanterä et al., 2014; Sasaki et al., 2004; Schultner & Pulliainen, 2020; Sundström et al., 1996), even if direct evidence for some of these is currently lacking in *Sclerodermus*.

Our model may apply to some further invertebrate taxa with sex ratio bias that also exhibit multi-foundress reproduction, parental and allo-parental care, and reproductive skew: candidates include social spiders (Grinsted & Lubin, 2019; Lubin & Bilde, 2007), although evidence of dominance is lacking (Grinsted & Lubin, 2019), and social thrips (Bono & Crespi, 2008; Gilbert et al., 2018), in which the absence of maturing eggs in many females may indicate possible reproductive suppression (Gilbert et al., 2018). Among vertebrates, the scenario we consider bears some similarities with empirical observations of offspring sex ratios of dominant and of subordinate mothers differing within primate groups that experience local resource competition (Silk, 1983), which can be seen as a more general statement of LMC (Hardy, 1997). For instance, in both macaques and spider monkeys, dominant or high-ranking mothers produce more of the “advantaged” sex that is more likely to achieve reproductive success within the group and subordinate or low-ranking mothers produce more of the “disadvantaged” sex that will typically disperse (Maestripieri, 2002; McFarland Symington, 1987; see also Silk, 1988, Schino, 2004). Verbal explanations (informal theory) for these findings have highlighted the importance of dominant females limiting the reproductive options of subordinates, for instance, via harassment and/or infanticide (e.g., van Schaik & Hrdy, 1991; Silk, 1983).

In contrast to *Sclerodermus*, the vast majority of parasitoid hymenopterans are socially solitary and very few gain benefits from their offspring sharing the resources of a given host with the offspring of other foundresses (Godfray, 1994; Hardy et al., 2013). While *Sclerodermus* reproduction via communally tended broods is unusually cooperative, current empirical and theoretical evidence suggests that it is also beset by the selfish behaviors of individual foundresses within groups.

## Supplementary Material

qrac005_suppl_Supplementary_Material

## Data Availability

No new data were used in this manuscript. All equations and simulation code are presented in the main text and supplementary information.
