# Supplementary material for: Individual- and group-level sex ratios under local mate competition: consequences of infanticide and reproductive dominance
Source: Evol Lett. 2023 Feb 9;7(1):13–23. doi: 10.1093/evlett/qrac005 (PMC10091503; doi:10.1093/evlett/qrac005)
Supplement: qrac005_suppl_Supplementary_Material [file qrac005_suppl_supplementary_material.pdf]

Supplementary Information

Individual and group level sex ratios under local mate competition: consequences of infanticide and reproductive dominance

Evolution Letters

Jussi Lehtonen[1*], Serena Malabusini[2], Xiaomeng Guo[3] and Ian C.W. Hardy[4]

[1]Department of Biological and Environmental Science, University of Jyväskylä, Jyväskylä, Finland, 40014

[2]Department of Food, Environmental and Nutritional Sciences (DeFENS), University of Milan, Italy

[3]College of Plant Protection, Nanjing Agricultural University, Nanjing, Jiangsu province, PR China

[4]Department of Agricultural Sciences, University of Helsinki, Helsinki, Finland, 00014

*Correspondence: jussi.lehtonen@iki.fi

Why direct aggression at sons of other foundresses?

While we do not aim to explain the coevolution of the sex ratio and dominance/aggressive behaviour, it is nevertheless instructive to consider on a general level why infanticide directed at sons might be advantageous, while infanticide directed at daughters is maladaptive.

Consider a local mate competition scenario where the evolving factor is infanticide, while sex ratio is fixed at *x*. We first examine a situation where there is no infanticide directed at sons, but a dominant foundress kills a proportion m of the daughters of (*n*-*d*) subordinate co-foundresses. Then fitness of the dominant foundress is:

$$w_d = (1 - x) + \frac{1}{n}[d(1 - x) + (1 - m)(n - d)(1 - x)] \qquad \text{(S1)}$$

Then $\frac{dw_d}{dm} = -\frac{n-d}{n}(1 - x)$ which is negative (assuming 0<*x*<1), i.e. infanticide of daughters of subordinate co-foundresses is maladaptive.

Now consider son-killing, directed at sons of (*n*-*d*) subordinate co-foundresses:

$$w_d = (1 - x) + \frac{x}{(dx+(1-m)(n-d)x)}[n(1 - x)] \qquad \text{(S2)}$$

where m is now the proportion of killed sons of subordinate co-foundresses. Now $\frac{dw_d}{dm} = \frac{n(n-d)}{\left(dm+n(1-m)\right)^2}(1 - x)$ which is positive (assuming 0<*x*<1), i.e. infanticide of sons of subordinate co-foundresses is adaptive. Note that this result depends on an assumption of all foundresses being fertilised even if males are scarce, i.e. the total number of fertilisations is proportional to $n(1 - x)$ regardless of the absolute number of males.

The intuitive explanation is simple: daughters of co-foundresses are potential mating partners for the focal individual's sons, whereas sons of co-foundresses are competitors for the focal individual's sons. Furthermore, even randomly directed male infanticide (i.e. including sons of the focal foundress) is selectively neutral under these assumptions: if the term (1-*m*) in equation (S2) affects all sons equally, including those of the focal foundress, it simply cancels out in the numerator and denominator of the fitness function. This suggests that the evolution of male infanticide can get started quite easily, even if discrimination of own sons from those of others is initially very inaccurate, and that subsequently improving discrimination would be adaptive.

Inclusive fitness analysis

While evolutionary game theory is perhaps the most powerful contemporary method to construct sex ratio models (West 2009), we can gain additional conceptual insight into the basic version of Model 2 (i.e. *d* dominant foundresses in a group of *n*) with a brief inclusive fitness analysis. Following (Taylor 1993; Denver and Taylor 1995; Frank 1998, p.195-197), consider the (marginal) inclusive fitness a mother gains from investing in an additional son when the population sex ratio is x^* (proportion sons):

$$\Delta W_{if}^{s} = \frac{d}{n}\frac{n(1-x^*)}{dx^*}(r_s - R_s^c)c_s + \frac{n-d}{d}0 = \frac{(1-x^*)}{x^*}(r_s - R_s^c)c_s \quad \text{(S3)}$$

and an extra daughter

$$\Delta W_{if}^{d} = r_d c_d + R_s^m c_s \quad \text{(S4)}$$

Let us consider the meaning of each term. The c_s and c_d terms are the class reproductive values for males and females and are unaltered by our model setup, so we will ignore them. Reading the first equation from left to right:

1) Reproduction via an extra son is realised only if the mother is a dominant, with probability $\frac{d}{n}$, analogous to decreased penetrance of a mutation altering the number of sons.

2) The penetrance effect is exactly cancelled out by the factor $\frac{n(1-x)}{dx}$, where daughters of *n* foundresses mate with sons of *d* foundresses, inflating the expected number of matings per surviving son.

3) We are left with relatedness coefficients. The first corresponds to the direct benefit from the extra son, so r_s is simply the relatedness of a foundress to her own sons.

4) R_s^c is more interesting: it is the average relatedness of a dominant mother to a random male in the patch competing for fertilisations (who must lose a mating for every mating her own extra son gains). When all mated females disperse to random patches, a mother's relatedness to sons that are not her own is 0, while her relatedness to her own sons remains r_s, resulting in average relatedness $R_s^c = \frac{r_s+0}{d} = \frac{r_s}{d}$. This is effectively a measure of realised local mate competition, hence the superscript *c*.

Now reading the second equation from left to right:

1) The extra daughter brings direct benefits weighted by relatedness r_d.

2) Further, the extra daughter provides additional mating opportunities to males in the patch. At conception, and without knowledge of which sons will survive, the daughter is equally likely to mate with any newly conceived male in the patch, so that $R_s^m = \frac{r_s+0}{n} = \frac{r_s}{n}$. This is effectively a measure of average sib-mating, hence the superscript *m*.

The central asymmetry that arises is that clustered (but random) mortality of sons can make R_s^c and R_s^m unequal.

Solving the equation $\Delta W_{if}^{s} = \Delta W_{if}^{d}$, we find

$$x^* = \frac{(r_s - R_s^c)c_s}{r_s c_s + r_d c_d - c_s R_s^c + c_s R_s^m} \tag{S5}$$

In a symmetric (haploid or diploid) genetic system where $c_d = c_s$ and $r_d = r_s$ the relatedness coefficients $R_s^c = \frac{r_s}{d}$ and $R_s^m = \frac{r_s}{n}$ reasoned above yield

$$x^* = \frac{1-\frac{1}{d}}{2-\frac{1}{d}+\frac{1}{n}} \tag{S6}$$

which is equation (5). The inclusive fitness perspective then provides an intuitive explanation for our results: a dominant foundress has inflated expected relatedness to a random competing male. Another way to view this is that clustered mortality increases local mate competition, but does not decrease average sib-mating. Equations (S5-S6) connect the current result to many prior results in sex ratio theory**.**

Equation (10) (pre-sex allocation dominance) has a very different interpretation but can also be derived with an inclusive fitness analysis. Intuitively it is clear that subordinates do not benefit from allocation into male offspring, and selection acts against this when allowed to evolve independently in subordinates, hence $x_s{}^* = 0$. This in turn implies that there are (*n*-*d*) subordinate females producing only daughters, which only the sons of *d* dominants compete for. Without these additional daughters, the dominants would evolve a standard LMC sex ratio of $\frac{(d-1)}{2d}$, but the 'free' extra daughters increase the *per capita* reproductive value of sons. We can rewrite the sex ratio of dominants as $x_d{}^* = \frac{(d-1)n}{2d^2} = \frac{(d-1)}{2d}(1 + \frac{n-d}{d})$: the additional allocation into sons by dominant foundresses is proportional to the relative number of additional daughters of subordinates that are available to the sons of dominants $(\frac{n-d}{d})$.. We can again perform an inclusive fitness analysis for dominant mothers:

$$\Delta W_{if}^{s} = \frac{d(1-x_d{}^*)+(n-d)(1)}{d x_d{}^*}(r_s - R_s^c)c_s \tag{S7}$$

$$\Delta W_{if}^{d} = r_d c_d + R_s^m c_s \tag{S8}$$

But now $R_s^c = R_s^m = \frac{1}{d}$, i.e. the measure of local mate competition equals the measure of sib-mating. Solving this obtains a generalised version of equation (10).

So, in the post-sex allocation dominance model the decoupling of local mate competition and sib-mating can be thought to drive the result. In the pre-sex allocation dominance model LMC and sib-mating both are reduced equally but sons of dominant foundresses have an additional pool of mating partners from the all-female subordinate clutches.

Model 2 when the distribution of reproductive potential can take any form

In the main text we derive a model where dominance is distributed in a simple way: only *d* foundresses produce sons, while subordinates are not allowed to do so. This reproductive skew could of course take other forms, where foundresses might have varying levels of dominance and their sons might have an arbitrary probability of survival. This situation is also analogous to one where the competitive ability of sons for matings varies from foundress to foundress. We use the term 'reproductive potential' as an umbrella term that captures both the survival of sons, and their relative competitive ability for matings. We derive an equation for Model 2 that accounts for a general distribution of reproductive potential over foundresses, such that this distribution is assumed to be similar in every group (analogous to the main model with *d* dominants per group).

Assume again that there are *n* foundresses per group. Each wild-type individual allocates a proportion $\hat{x}$ of its reproductive resources into sons and the rest into daughters (a constant multiplicative factor indicating the absolute number of offspring does not affect the results and is omitted). Now assume that each foundress may have their reproductive output via sons altered by a multiplicative coefficient q_k where *k*=1,2,...*n*, accounting for the reproductive potential of the sons of foundresses 1 through to *n*. For example, the coefficients $q_1 = q_2 = 0$ and $q_{3=}q_{4=\dots} = q_n = 1$ can be interpreted such that two foundresses have all their sons killed (or otherwise prevented from mating) while the rest have sons normally. But the coefficients can in principle take any non-negative values, although values in the range [0,1] have the most natural biological interpretation as a diminished survival or diminished reproductive competitiveness of sons. The set of values $\{q_1, q_2, \dots, q_n\}$ is assumed to be the same for each group, and within a group the values are assigned to the foundresses randomly. In other words, the foundresses experience the *q* values randomly with respect to their phenotype and genotype. The process could be truly random or could correspond to some external factor which is independent of foundress characteristics. Then, any given foundress is assigned a coefficient q_k with probability 1/*n*. We will denote the sum of the q_k values with $d = \sum_{k=1}^{n} q_k$. The expected fitness of a rare mutant is then

$$w = (1 - x) + \sum_{k=1}^{n} \left(\frac{1}{n} \frac{q_k x}{q_k x + (d - q_k)\hat{x}}\right) \left((1 - x) + (n - 1)(1 - \hat{x})\right) \quad \text{(S9)}$$

The direction of selection is determined by the derivative

$$\left.\frac{dw}{dx}\right|_{x=\hat{x}} = -1 - \sum_{k=1}^{n} \left(\frac{1}{n} \frac{q_k}{d}\right) + n(1 - x) \left(\sum_{k=1}^{n} \left(\frac{1}{n} \frac{q_k}{xd}\right) - \sum_{k=1}^{n} \left(\frac{1}{n} \frac{x {q_k}^2}{x^2 d^2}\right)\right) = -1 - \frac{1}{n} + n(1 - x) \left(\frac{1}{nx} - \frac{1}{x} \sum_{k=1}^{n} \frac{{q_k}^2}{nd^2}\right) \quad \text{(S10)}$$

The variance in q is $\sigma^2 = \sum_{k=1}^{n} \frac{{q_k}^2}{n} - \left(\sum_{k=1}^{n} \frac{q_k}{n}\right)^2 = \sum_{k=1}^{n} \frac{{q_k}^2}{n} - \left(\frac{d}{n}\right)^2$, so $\sum_{k=1}^{n} \frac{{q_k}^2}{n} = \sigma^2 + \left(\frac{d}{n}\right)^2$ and $\sum_{k=1}^{n} \frac{{q_k}^2}{nd^2} = \frac{(\sigma^2 + \left(\frac{d}{n}\right)^2)}{d^2} = \frac{1}{n^2} + \frac{1}{n^2}\left(\frac{\sigma}{d/n}\right)^2 = \frac{1}{n^2}(1 + \mathrm{c}_\mathrm{v}^2)$, where $\frac{\sigma}{d/n} = \mathrm{c}_\mathrm{v}$ is the coefficient of variation.

Therefore, we can find the candidate ESS sex ratio by solving

$$\left.\frac{dw}{dx}\right|_{x=\hat{x}} = -1 - \frac{1}{n} + n(1-x)\left(\frac{1}{nx} - \frac{1}{xn^2}(1 + {\mathrm{c}_\mathrm{v}}^2)\right) = 0 \qquad \text{(S11)}$$

which obtains

$$x^* = \frac{1 - (1 + {\mathrm{c}_\mathrm{v}}^2)/n}{2 - {\mathrm{c}_\mathrm{v}}^2/n} \qquad \text{(S12)}$$

As an example of this more general result, equation (5) can be recovered by computing the coefficient of variation (or equivalently, directly computing the squared coefficient of variation ${\mathrm{c}_\mathrm{v}}^2$) corresponding to the distribution where *d* individuals contribute equally to making all the sons (with reproductive potential *q*) while the *n*-*d* remaining individuals make no sons (reproductive potential 0).

The mean reproductive potential is then $\frac{dq}{n}$, and the variance in reproductive potential $\frac{dq^2}{n} - \left(\frac{dq}{n}\right)^2$. Therefore, the squared coefficient of variation is ${\mathrm{c}_\mathrm{v}}^2 = \left(\frac{dq^2}{n} - \left(\frac{dq}{n}\right)^2\right) / \left(\frac{dq}{n}\right)^2 = n/d - 1$. Substituting this expression into $x^* = \frac{1-(1+{\mathrm{c}_\mathrm{v}}^2)/n}{2-{\mathrm{c}_\mathrm{v}}^2/n}$ we find $x^* = \frac{1-\left(1+\frac{n}{d}-1\right)/n}{2-(\frac{n}{d}-1)/n} = \frac{1-1/d}{2-1/d-1/n}$.

Haplodiploid models

Model 1 is equivalent to a classic LMC model, for which haplodiploid results have been derived previously (Hamilton 1979; West 2009). Here, we derive haplodiploid results for Models 2 and 3. Most of the relevant equations can be found, for example, on p. 319 of Frank (1986) and Box 1 of Gardner and Hardy (2020) (with further details in references therein). For Model 2, assume the level of sibmating (West 2009, references on p. 80) is k. Then the inbredness of a focal foundress (*i.e.*, consanguinity of her parents) is $f = k/(4-3k)$. Under haplo-diploidy, the consanguinity of a mother to her daughters is $p_d = (1 + 3f)/4$, and the consanguinity of a mother to her sons is $p_s = (1 + f)/2$. We are interested in the relative 'valuation' of daughters over sons in terms of how much more closely related the mother is to her daughters than to her sons. Calculating p_d/p_s by substituting $f = k/(4-3k)$ we find that $p_d/p_s = 1/(2-k)$. Finally, under haplo-diploidy, the class reproductive value of females is twice that of males (Hamilton 1972; Bulmer 1994; Taylor and Frank 1996), so we further multiply the expression by 2 and obtain

$2p_d/p_s = 2/(2-k)$.

The Model 2 components corresponding to daughters in Equation 4 in the main text ($\left(\frac{d}{n}\right)(1-x)$ and $\left(\frac{n-d}{n}\right)(1-x)$) are therefore multiplied by the factor $2/(2-k)$ and the calculation is otherwise repeated in the same manner.

Solving the resulting equation, we find the evolutionarily stable primary sex ratio

$$x^* = \frac{(d-1)(2-k)n}{dn(4-k)+(2-k)(d-n)} \quad \text{(S13)}$$

Computing the group-level sex ratio corresponding to (S13) when all sons of dominants survive, following the main text, obtains

$$x^*_{group} = \frac{(d-1)(2-k)}{d(2-k)+2n} \quad \text{(S14)}$$

In the simple situation where the number of foundresses is always n within all foundress groups throughout the population, we have $k = 1/n$. This applies even in the current model where some foundresses do not have any sons. Consider a female that is the offspring of a random foundress in a patch with n foundresses. With probability d/n her mother is a dominant foundress, and she therefore has brothers in the patch. Now consider a random male in the same patch, with whom the focal female mates. Males are born to d foundresses, and therefore, conditional on the focal female being the daughter of a dominant female, the probability that the focal male has the same mother as the focal female is $1/d$. The overall probability of a random daughter pairing with her brother is then $d/n*1/d = 1/n$, and this is also the expected fraction of sibmating, k, and (S13) becomes

$$x^* = \frac{(d-1)(2n-1)n}{n(1-2n)+d(4n^2+n-1)} \quad \text{(S15)}$$

Similarly, (S14) becomes

$$x^*_{group} = \frac{(d-1)(2n-1)}{d(2n-1)+2n^2} \qquad \text{(S16)}$$

Model 3 is more complex because now sibmating, *k,* depends on the current sex ratio. If dominants produce a fraction *x* of sons, then the number of daughters of dominants in the patch is proportional to (1-*x*)*d*, while the total number of daughters is proportional to (1-*x*)*d* + (*n*-*d*). Therefore, the probability that a randomly chosen daughter in a patch has a mother who is a dominant is $\frac{(1-x)d}{(1-x)d\,+\,(\mathrm{n-d})} = \frac{(1-x)d}{n-xd}$. The probability that any one of these daughters will mate with one of their brothers is $1/d$, and therefore the expected fraction of overall sibmating is $\frac{(1-x)}{n-xd}$. From here we can repeat the calculations, as above, and find

$$2\,p_d/p_s = 2\,\frac{n-dx}{2n+x-2dx-1}$$

Now we can solve for the primary sex ratio as above, while noting that contrary to Model 2, *x* now appears in the relatedness coefficient above.

Noting the upper limit of 1, the solution is

$$x^* = \min\left(1, \frac{a-\sqrt{\mathrm{b}}}{c}\right) \qquad \text{(S17)}$$

where

$$a = n - 3dn + d^2(-1+6n)$$

$$b = n^2 - 6dn^2 + 2d^3n(-7+2n) + d^4(1+2n)^2 + d^2n(2+13n)$$

$$c = 2d^2(-1+4d).$$

For the group-level sex ratio when all sons of dominants survive, we have

$x^*_{group} = \min\left(\frac{d}{n}, \frac{d}{n}\left[\frac{a-\sqrt{\mathrm{b}}}{c}\right]\right)$, with *a*, *b* and *c* as above. (S18)

Presenting the sex ratio for Model 3 with *k* as a free parameter as in Model 2 is not useful, because *k* depends on the sex ratio itself.

We can perform some simple checks for consistency with earlier models:

Substituting *d*=*n* into Equation (S13) or (S14) we find

$$x^* = \frac{(n-1)(2-k)}{n(4-k)} \tag{S19}$$

which is Equation (4.3) in West (2009) for haplodiploids with no dominance structure.

Substituting *d*=*n* into any of the equations (S15) to (S18) yields

$$x^* = \frac{(n-1)(2n-1)}{n(4n-1)} \tag{S20}$$

which is Equation (4.2) West (2009) for haplodiploids with no dominance structure and constant group size *n*.

Comparison of haplodiploid and diploid results

The haplodiploid equations presented in the previous section are considerably more complicated and less intuitive than the diploid results. Figures S1 and S2 indicate that differences between results of diploid and haploid models are minor, and likely smaller than error in empirical measurement. Furthermore, the diploid model already demonstrates the salient conceptual differences compared to earlier LMC models, while haplodiploidy requires a small additional correction in the previous models as well as the current ones. We therefore focus on the diploid model in the main text for expositional clarity. It is nevertheless important to be aware of the difference that the genetic system can make in these models, although, in fact, the difference between the diploid and haplodiploid version of Models 2 and 3 is quite small (Figures S1 and S2), and likely to be empirically indistinguishable.

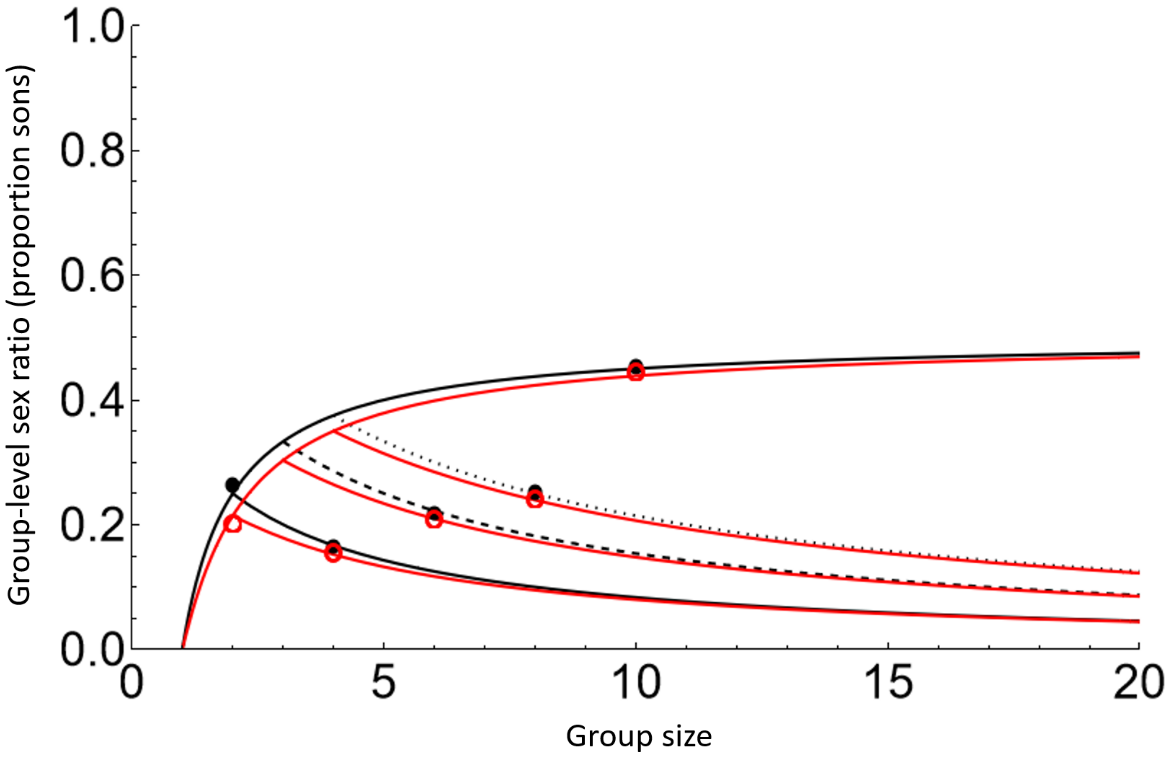

Figure S1. Comparison of haplodiploid (Equation S16) and diploid results (Equation 8, main text) for Model 2. Results in black are exactly as in Figure 3 in the main text. Results in red are haplodiploid equivalents of Model 2, the open red circles being haplodiploid simulations (see below).

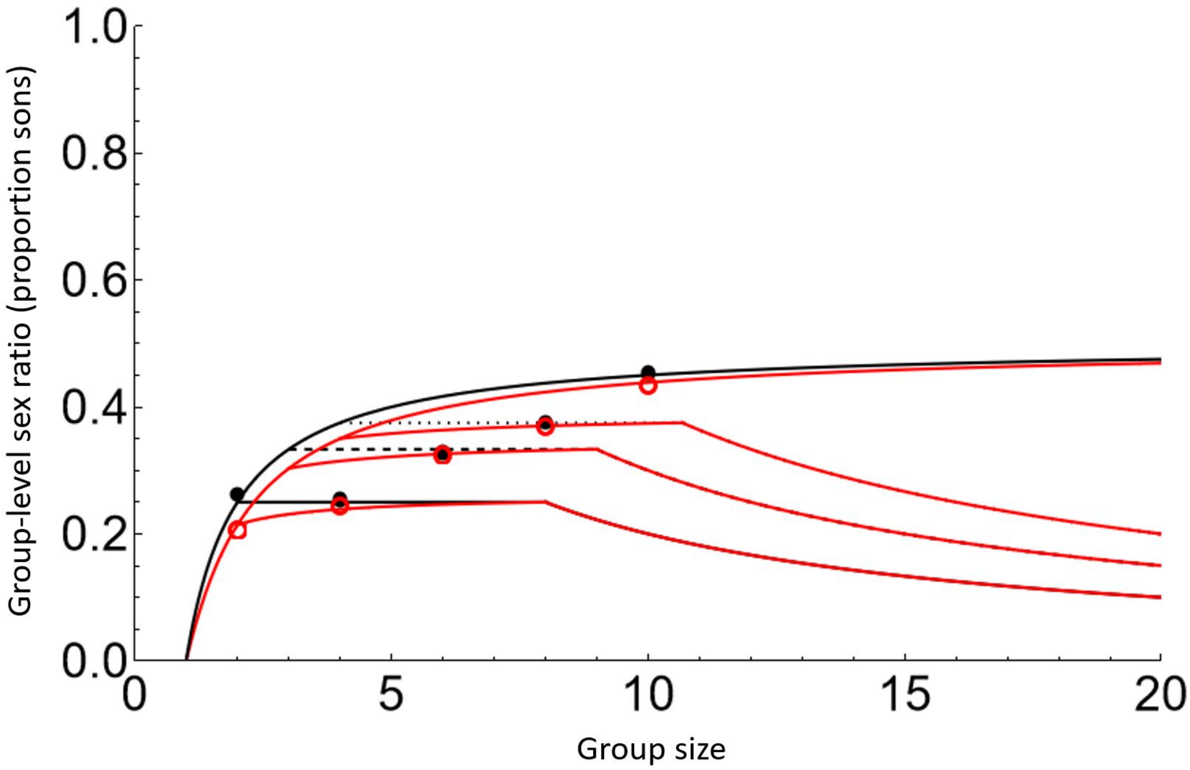

Figure S2. Comparison of haplodiploid (Equation S18) and diploid results (Equation 15, main text) for Model 3. See the legend to Figure S1 for further explanation.

Simulations

The validity of the analytical results for Models 2-3 was confirmed using individual-based simulations (see code below), as seen in Figures S1 and S2 above. Simulations also function as informal stability analyses for the equilibria. Below we present more extensive simulation results for the diploid and haplodiploid versions of Model 3, which is more complex than Model 2 in that the dominant and subordinate sex ratios evolve independently (although there is coevolution between them).

In each figure below, the top panel shows values of the evolving sex ratio for dominants, for subordinates, and for the population mean (which here is equivalent to the mean group-level sex ratio). The bottom panel shows the genetic variance in the dominant and subordinate sex ratios. Note that in some panels the number of dominants equals the total number of foundresses, so that there are no subordinates. In such cases the genes for the subordinate sex ratio strategy are not expressed, and hence the trait drifts, as does its variance. Contrarily, the fact that variance decreases deterministically when traits are expressed informally indicates that the equilibria are evolutionarily stable (Lehtonen 2018), while the fact that the traits evolve towards these equilibria informally indicates that they are convergence stable (Otto and Day 2007).

In each panel, the total number of groups is 500 (see Figure S3H for an exception), foundress brood size at oviposition is 40 (a realistic number: see e.g. Fig. 6 in (Abdi et al. 2020)), the probability of a new mutation at conception is 0.00001 per allele, and mutations are picked from a normal distribution with mean 0 and standard deviation 0.1. That is, mutations change the sex ratio by a random amount, which is restricted such that sex ratio cannot vary outside the range [0,1]. Note that this allows arbitrarily large mutations. Alleles combine in an additive manner to produce phenotypes. Each simulation was run for 2000 generations, starting from randomly chosen alleles (i.e. each allele in the population was initially given a random value). Open circles indicate analytical predictions. Group size, number of dominants, ploidy, and the ability for plastic response to dominance status (with 1 indicating that a plastic response is possible) are indicated at the top of each panel. Red lines indicate dominant sex ratios, blue lines indicate subordinate sex ratios, and black lines indicate group or population average sex ratios. Note that when all foundresses are dominants, the dominant and group sex ratios overlap, and the subordinate sex ratio drifts freely because it is not exposed to selection.

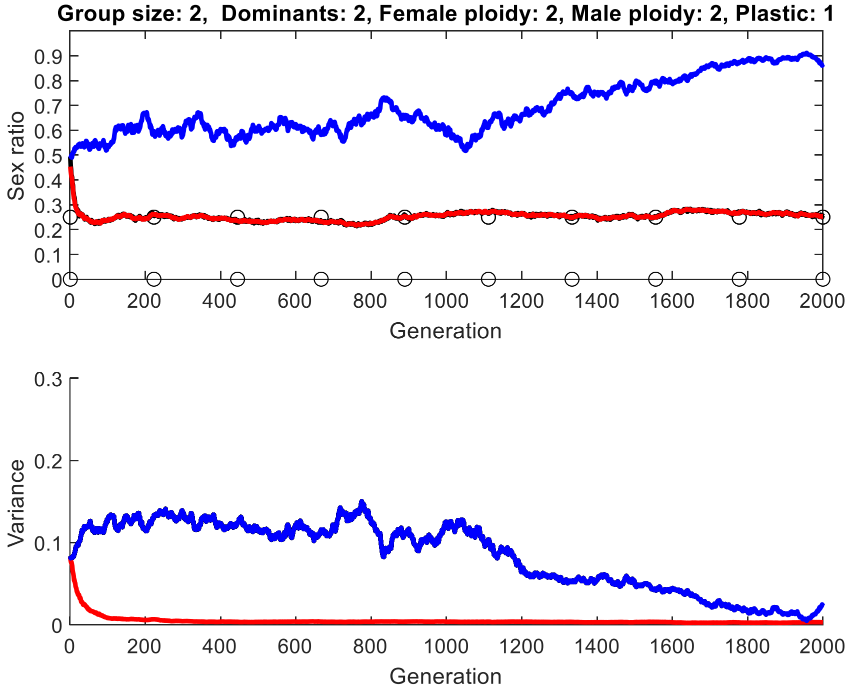

Figure S3A. Here all individuals are dominants, so group sex ratios and dominant sex ratios overlap, and the subordinate sex ratio drifts freely because it is not exposed to selection.

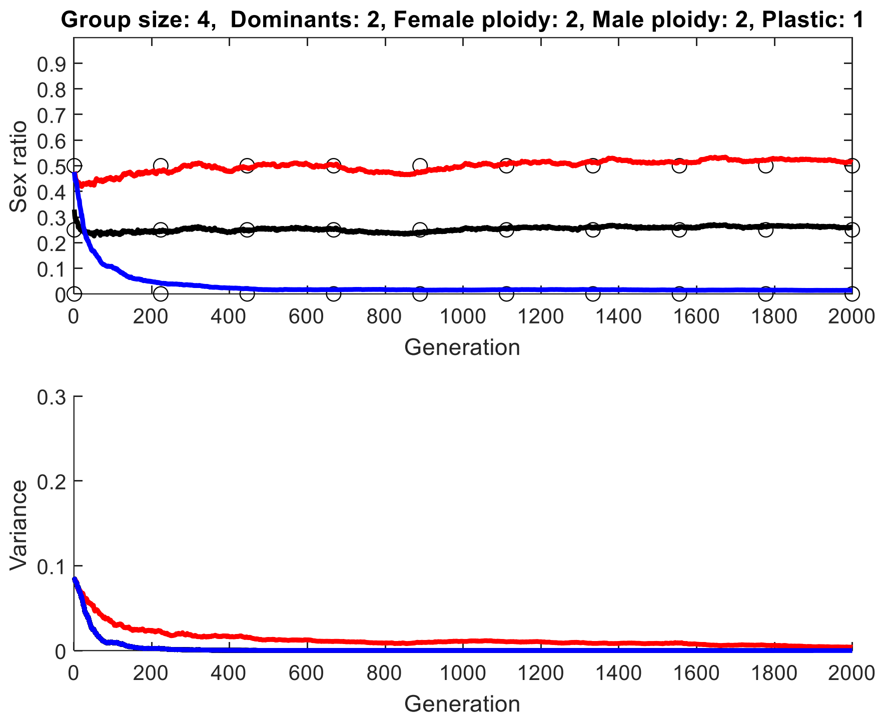

Figure S3B. Now both dominant and subordinate sex ratios are exposed to selection. Subordinate sex ratios go to 0, and all sex ratios fit analytical predictions. Genetic variance decreases around the equilibria, informally indicating evolutionary stability.

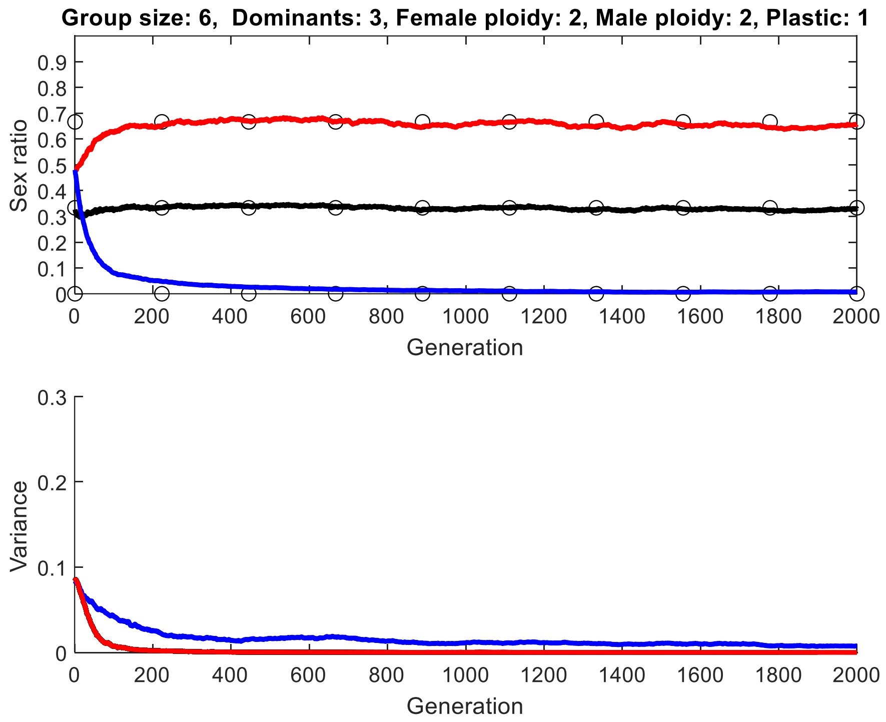

Figure S3C. See legend to S3B.

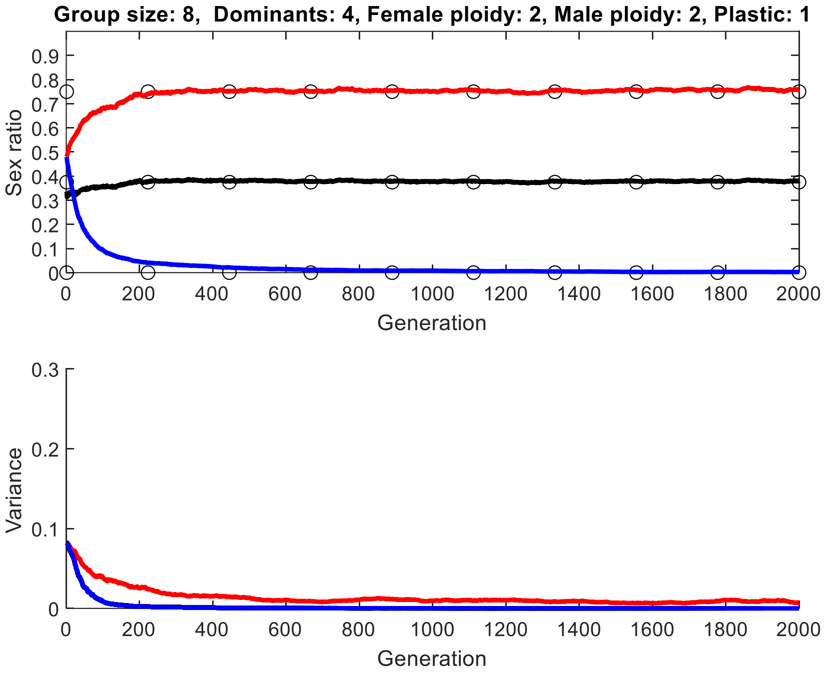

Figure S3D. See legend to S3B.

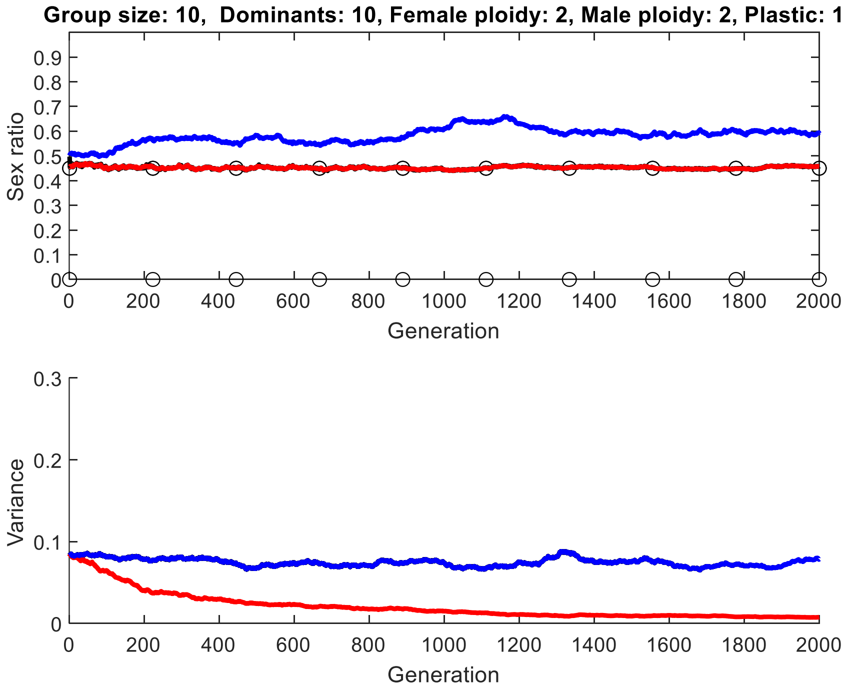

Figure S3E. Again, all individuals are dominants, so group sex ratios and dominant sex ratios overlap, and the subordinate sex ratio drifts freely because it is not exposed to selection.

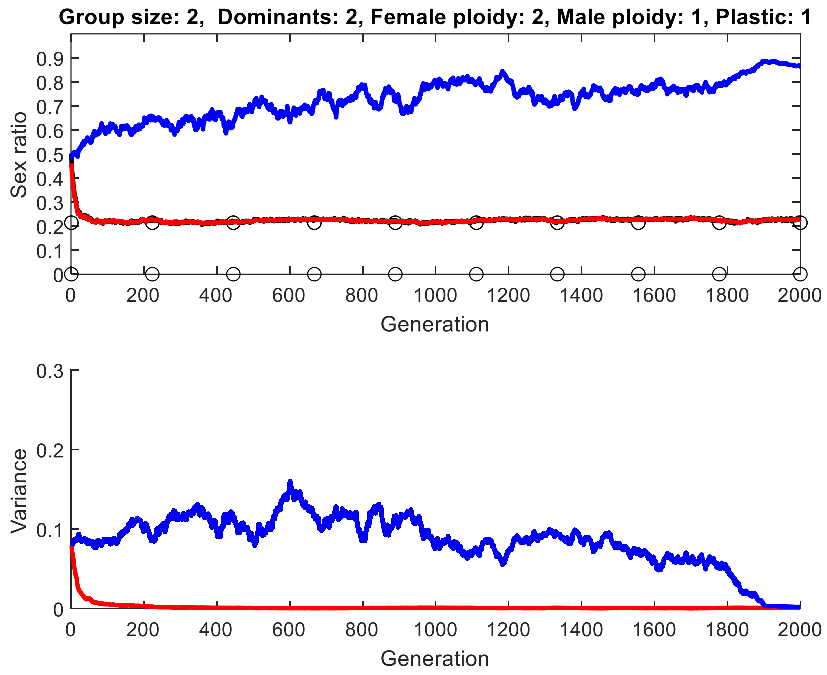

Figure S3F. As S3A, but with a haplodiploid genetic system.

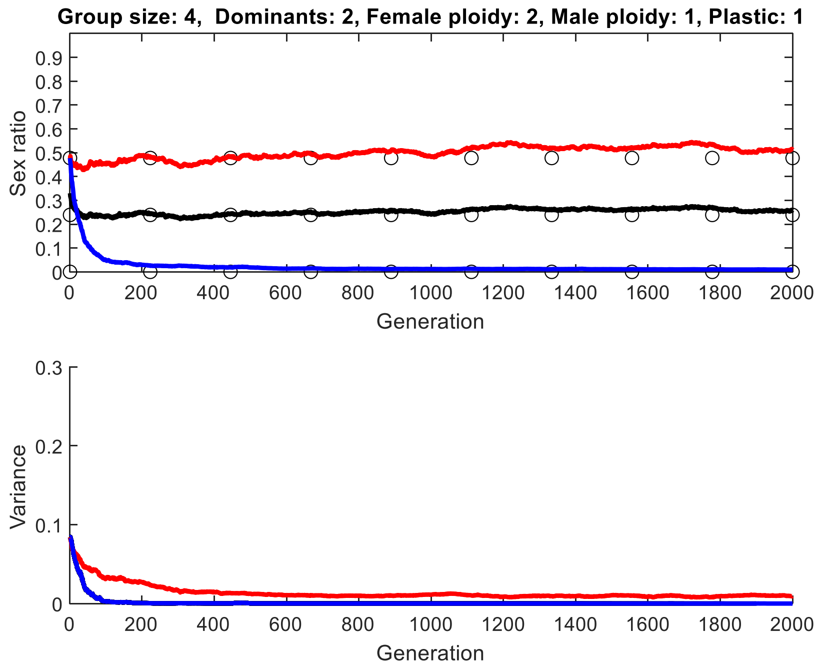

Figure S3G. As S3B, but with haplodiploid genetic system. This panel was simulated again (Figure S3H below) with the number of groups increased tenfold to re-investigate the potential discrepancy between the analytical prediction and the simulation result (latter half of red line).

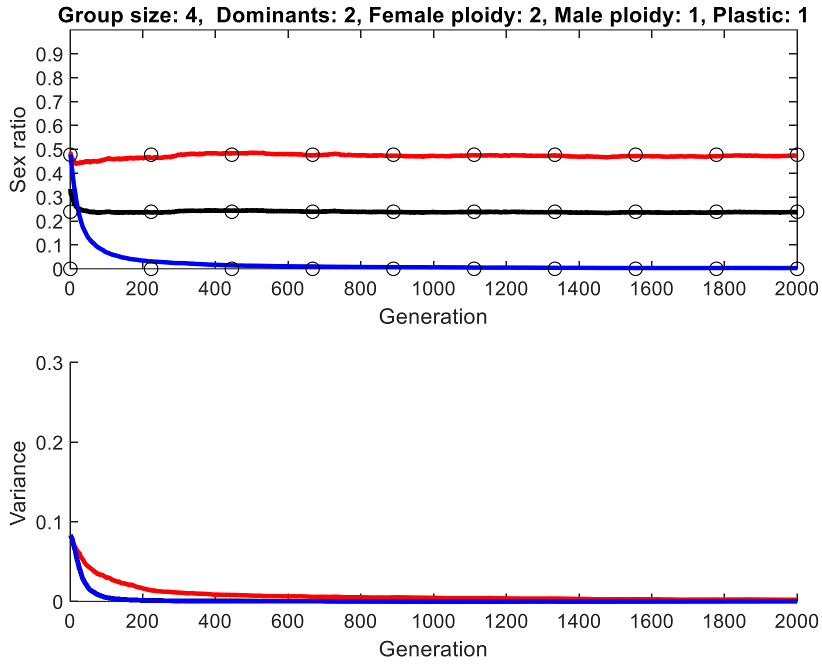

Figure S3H. As S3G, but with 5000 groups instead of 500. With a larger population size the simulation seems to match the analytical results better than in S3G above (simulating all results using such a large number of groups would be prohibitively slow, particularly with larger group sizes).

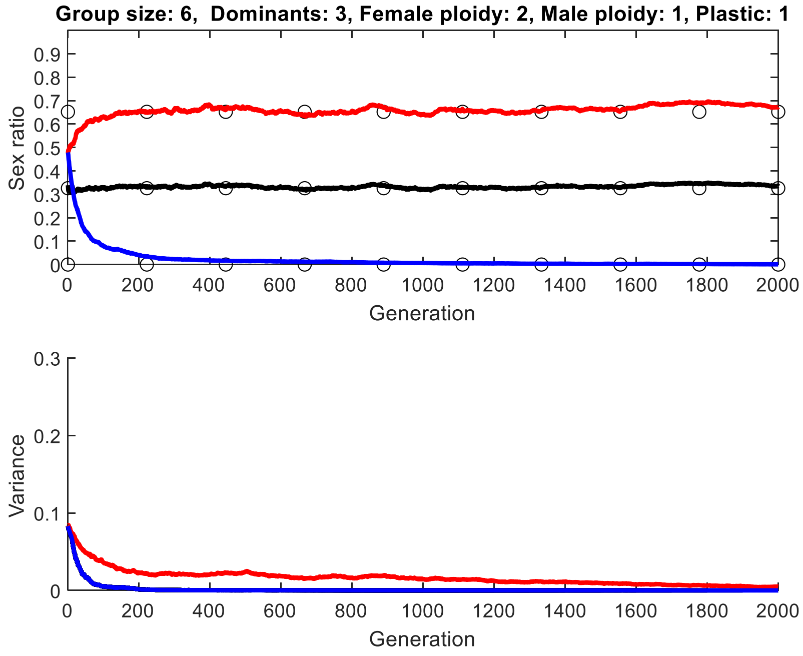

Figure S3I. As S3C, but with haplodiploid genetic system.

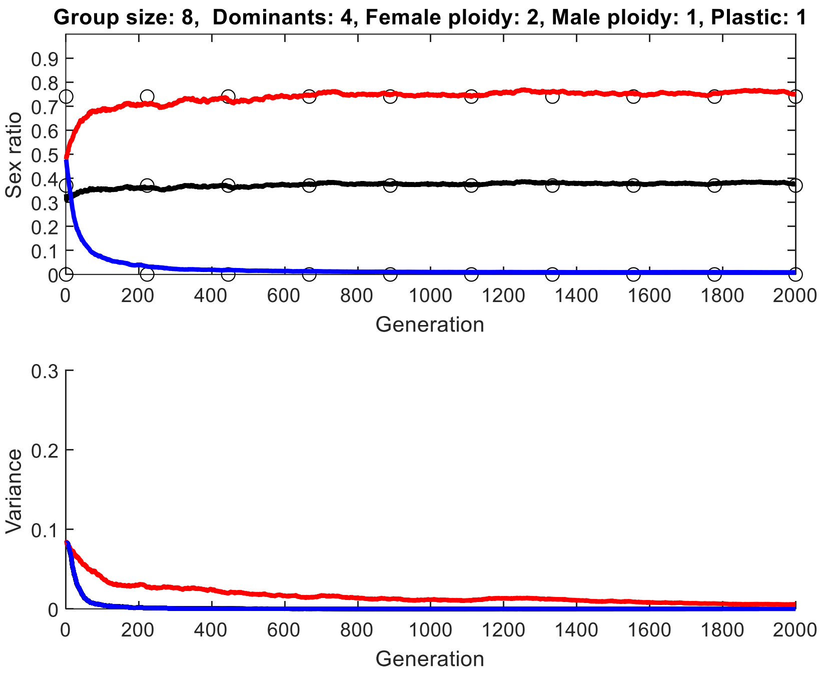

Figure S3J. As S3D, but with haplodiploid genetic system.

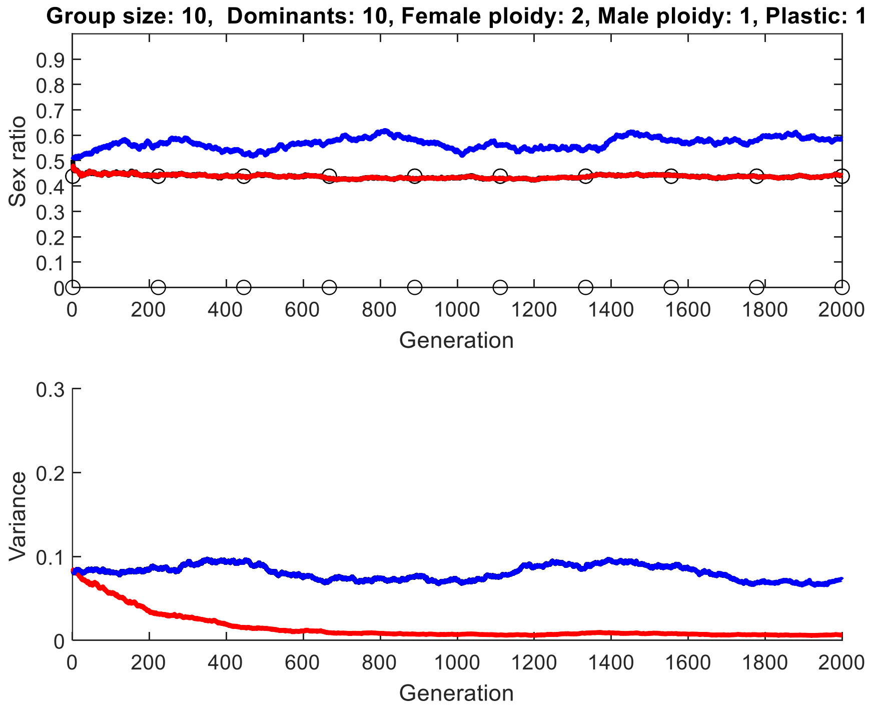

Figure S3K. As S3E, but with haplodiploid genetic system.

Simulation code (matlab)

```
function SexRatioDominance(
groupsize,dominants,groupnumber,clutchsize,maxtime,femaleploidy,maleploidy,respondtostatus,randomstart,mutrate,stdev)
%groupsize=number of foundresses per group
%dominants=number of dominant foundresses per group
%groupnumber=number of groups in the global population
%clutchsize=total number of offspring conceived per foundress
%maxtime=number of generations the simulation is run for
%femaleploidy=ploidy of females (set to 2)
%maleploidy=ploidy of males (set to 1 or 2)
%respondtostatus=ability for plastic response to dominance status
%randomstart=start with simulation with randomly picked alleles
%mutrate=probability of mutation per allele per generation
%stdev=standard deviation of mutation distribution


G=groupnumber;
femalematrixsize=femaleploidy*(respondtostatus+1)+maleploidy*(respondtostatus+1);
zeros(groupsize,femalematrixsize);
timeforaverage=100;
hist_currentpopmean=zeros(timeforaverage,1);
hist_currentindmean=zeros(timeforaverage,1);
hist_currentdommean=zeros(timeforaverage,1);
if respondtostatus==1
    hist_currentsubmean=zeros(timeforaverage,1);
end

d=dominants; n=groupsize;
if femaleploidy==maleploidy
    if respondtostatus==0
        individualprediction=((d-1)*n)/(d-n+2*d*n);
        patchprediction=(d-1)/(d+n);
    else
        individualprediction=min(1,((d-1)*n)/(2*d^2 ));
        patchprediction=min(d/n,((d-1))/(2*d));
    end
end
if and(femaleploidy==2,maleploidy==1)
    if respondtostatus==0
        individualprediction=((d-1)*n*(2*n-1))/(d*(4*n^2+n-1)+n-2*n^2 );
        patchprediction=((d-1)*(2*n-1))/(d*(2*n-1)+2*n^2 );
    else
        individualprediction=min(1, (n - 3*d *n + d^2 *(-1 + 6* n) - sqrt(  n^2 - 6* d *n^2 + 2* d^3* n *(-7 + 2* n) + d^4* (1 + 2*
n)^2+d^2* n *(2 + 13* n)))/(2*d^2 *(-1 + 4 *d)));
        patchprediction=d*individualprediction/n;
    end
end

clear('d'); clear('n');



clear('femalealleles');
clear('malealleles');
for group=1:G
    for i=1:groupsize
        if randomstart==1
            femalealleles{group}=rand(groupsize,femalematrixsize);
        else
            femalealleles{group}=0.5*ones(groupsize,femalematrixsize);
        end
        femalealleles{group}=min(1,max(0,femalealleles{group}));
    end
end





historysr=[];
historyvar=[];
historymin=[];
historymax=[];
popsr=-1;

for t=1:maxtime




    updates=100;
    if or(t==1, t/updates==floor(t/updates))
        [t maxtime  t/maxtime];
    end



    totaldaughtercounter=0;
    totalsoncounter=0;
    allfertiliseddaughters=[];

    for group=1:G

        howmanyfoundresses=size(femalealleles{group},1);
        dominantlabels=randsample(howmanyfoundresses,dominants);
```

```
        daughtercounter=0;
        soncounter=0;
        daughtergenes=[];

        for mother=1:howmanyfoundresses
            dominancestatus=~isempty(intersect(mother,dominantlabels));
            if dominancestatus==0
            end
            if dominancestatus==1
                sexratio=mean(femalealleles{group}(mother,1:femaleploidy));
            else
                if respondtostatus==0
                    sexratio=mean(femalealleles{group}(mother,1:femaleploidy));
                end
                if respondtostatus==1
                    sexratio=mean(femalealleles{group}(mother,(femaleploidy+1):(2*femaleploidy)));
                end
            end

            for j=1:clutchsize
                maternalalleles=[];
                paternalalleles=[];
                trait1=[];
                trait2=[];

                maternalalleles(1)=femalealleles{group}(mother,(ceil(femaleploidy*rand)));
                if rand<mutrate
                    maternalalleles(1)=mutatesr(maternalalleles(1),stdev);
                end
                paternalalleles(1)=femalealleles{group}(mother,(femaleploidy*(1+respondtostatus)+ceil(maleploidy*rand)));
                if rand<mutrate
                    paternalalleles(1)=mutatesr(paternalalleles(1),stdev);
                end

                if respondtostatus==1
                    maternalalleles(2)=femalealleles{group}(mother,(femaleploidy+ceil(femaleploidy*rand)));
                    if rand<mutrate
                        maternalalleles(2)=mutatesr(maternalalleles(2),stdev);
                    end
                    paternalalleles(2)=femalealleles{group}(mother,(femaleploidy*(1+respondtostatus)+maleploidy+ceil(maleploidy*rand)));
                    if rand<mutrate
                        paternalalleles(2)=mutatesr(paternalalleles(2),stdev);
                    end
                end


                trait1=[maternalalleles(1) paternalalleles(1)];
                if respondtostatus==1
                    trait2=[maternalalleles(2) paternalalleles(2)];
                end


                whichsex=rand;

                if whichsex>sexratio
                    daughtercounter=daughtercounter+1;
                    daughtergenestemp=datasample(trait1,femaleploidy,'replace',false);
                    if respondtostatus==1
                        daughtergenestemp=[daughtergenestemp datasample(trait2,femaleploidy,'replace',false)];
                    end
                    daughtergenes(daughtercounter,:)=[daughtergenestemp mother];

                end

                if and(dominancestatus==1,whichsex<sexratio)
                    soncounter=soncounter+1;
                    if and(maleploidy==1,femaleploidy==2)
                        songenestemp=maternalalleles;
                    end
                    if and(maleploidy==1,femaleploidy==1)
                        songenestemp=datasample(trait1,maleploidy,'replace',false);
                        if respondtostatus==1
                            songenestemp=[songenestemp datasample(trait2,maleploidy,'replace',false)];
                        end
                    end
                    if maleploidy==2
                        songenestemp=[trait1];
                        if respondtostatus==1
                            songenestemp=[trait1 trait2];
                        end
                    end


                    songenes(soncounter,:)=[songenestemp mother];

                end

            end

        end
        if and(daughtercounter>0,soncounter>0)
            for femalepartner=1:daughtercounter
                malepartner=ceil(rand*soncounter);

daughtergenes(femalepartner,(femaleploidy*(1+respondtostatus)+1):(femaleploidy*(1+respondtostatus)+maleploidy*(1+respondtostatus)))=song
enes(malepartner,1:maleploidy*(1+respondtostatus));
            end

            allfertiliseddaughters=[allfertiliseddaughters; daughtergenes];
```

```
    end

    totaldaughtercounter=totaldaughtercounter+daughtercounter;
    totalsoncounter=totalsoncounter+soncounter;

end


foundresscandidates=1:size(allfertiliseddaughters,1);
for group=1:G
    femalealleles{group}=[];
    [foundressesthisgroup,idx]=datasample(foundresscandidates,groupsize,'replace',false);
    femalealleles{group}=allfertiliseddaughters(foundressesthisgroup,:);
    foundresscandidates(idx)=[];
end


currentpopmean=totalsoncounter/(totalsoncounter+totaldaughtercounter);
currentindmean=mean(mean(allfertiliseddaughters));
currentdommean=mean(mean(allfertiliseddaughters(:,1:femaleploidy)));
currentdomvar1=var(mean(allfertiliseddaughters(:,1:femaleploidy),2),1);
currentdomvar2=var(allfertiliseddaughters(:),1);
if femaleploidy==1
    currentdomvar3=var(allfertiliseddaughters(:,1),1);
end
if femaleploidy==2
    currentdomvar3=var([allfertiliseddaughters(:,1); allfertiliseddaughters(:,2)] ,1);
end
if respondtostatus==1
    currentsubmean=mean(mean(allfertiliseddaughters(:,(femaleploidy+1):2*femaleploidy)));
    if femaleploidy==1
        currentsubvar3=var(allfertiliseddaughters(:,(femaleploidy+1)),1);
    end
    if femaleploidy==2
        currentsubvar3=var([allfertiliseddaughters(:,(femaleploidy+1)); allfertiliseddaughters(:,2*femaleploidy)] ,1);
    end
end

fullhist_popmean(t)=currentpopmean;
fullhist_indmean(t)=currentindmean;
fullhist_dommean(t)=currentdommean;
fullhist_domvar(t)=currentdomvar3;


hist_currentpopmean(end)=currentpopmean;
hist_currentpopmean=circshift(hist_currentpopmean,1);
hist_currentindmean(end)=currentindmean;
hist_currentindmean=circshift(hist_currentindmean,1);
hist_currentdommean(end)=currentdommean;
hist_currentdommean=circshift(hist_currentdommean,1);
if respondtostatus==1
    fullhist_submean(t)=currentsubmean;
    fullhist_subvar(t)=currentsubvar3;
    hist_currentsubmean(end)=currentsubmean;
    hist_currentsubmean=circshift(hist_currentsubmean,1);
end



updates=100;
if or(t==1, t/updates==floor(t/updates))


    [patchprediction currentpopmean currentpopmean-patchprediction]
    if respondtostatus==1
        [individualprediction mean(hist_currentindmean) mean(hist_currentdommean) mean(hist_currentsubmean)]
    end
    if respondtostatus==0
        [individualprediction mean(hist_currentindmean) mean(hist_currentdommean)]
    end
    t
    if respondtostatus==0
        [currentpopmean currentdommean]
    end
    if respondtostatus==1
        [[patchprediction; currentpopmean] [individualprediction; currentdommean] [0;currentsubmean]]
        [[patchprediction; currentpopmean] [individualprediction; currentdommean] [0;currentsubmean]]
    end

    [patchprediction individualprediction 0]
    [mean(fullhist_popmean(ceil(t/2):t)) mean(fullhist_dommean(ceil(t/2):t)) currentdomvar2]

    if respondtostatus==0
        figure(1);
        subplot(211);
        plot(linspace(1,t,10),patchprediction*ones(size(linspace(1,t,10))),'ok');
        hold on;
        plot(linspace(1,t,10),individualprediction*ones(size(linspace(1,t,10))),'ok');
        subplot(211);
        plot(fullhist_popmean,'k');
        plot(fullhist_dommean,'k');
        axis([0 t 0 1]);
        drawnow;
        hold off;
        subplot(212); hold on;
        plot(1*fullhist_domvar,'k');
        axis([0 t 0 0.1]);
        drawnow;
       hold off;
```

```
        end

        if respondtostatus==1
            figure(1);
            subplot(211);
            plot(linspace(1,t,10),patchprediction*ones(size(linspace(1,t,10))),'ok');
            hold on;
            plot(linspace(1,t,10),individualprediction*ones(size(linspace(1,t,10))),'ok');
            plot(linspace(1,t,10),zeros(size(linspace(1,t,10))),'ok');
            subplot(211);
            plot(fullhist_popmean,'k');
            plot(fullhist_dommean,'k');
            plot(fullhist_submean,'k');
            axis([0 t 0 1]);
            drawnow;
            hold off;
            subplot(212); hold on;
            plot(1*fullhist_domvar,'k');
            plot(1*fullhist_subvar,'k','Linewidth',2);
            axis([0 t 0 0.3]);
            drawnow;
            hold off;
        end

        figure(1); subplot(211);
        figtitle=strcat("Group size: ",num2str(groupsize),",  Dominants: ", num2str(dominants), ", Female ploidy: ",
num2str(femaleploidy),", Male ploidy: ", num2str(maleploidy), ", Plastic: ", num2str(respondtostatus));
        title(figtitle);
        xlabel('Generation');
        ylabel('Sex ratio');
        subplot(212);
        xlabel('Generation');
        ylabel('Variance');

        drawnow;
    end

end
```

Function for mutations for the above simulation (matlab)

```
function [mutated] = mutatesr(wildtype, stdev)
%This function enables easy alteration of mutation distribution

mutated=min(1,max(0,wildtype+normrnd(0,stdev)));

end
```

References for supplementary information

Abdi, M. K., I. C. W. Hardy, C. Jucker, and D. Lupi. 2020. Kinship effects in quasi-social parasitoids II: co-foundress relatedness and host dangerousness interactively affect host exploitation. Biol. J. Linnean Soc. 130:642-660.

Bulmer, M. G. 1994. Theoretical evolutionary ecology. Sinauer Associates, Sunderland, Massachusetts.

Denver, K. and P. D. Taylor. 1995. An inclusive fitness model for the sex ratio in a partially sibmating population with inbreeding cost. Evol. Ecol. 9:318-327.

Frank, S. A. 1986. Hierarchical selection theory and sex ratios I. General solutions for structured populations. Theoretical Population Biology 29:312-342.

Frank, S. A. 1998. Foundations of social evolution. Princeton University Press, Princeton, New Jersey.

Gardner, A. and I. C. W. Hardy. 2020. Adjustment of sex allocation to co-foundress number and kinship under local mate competition: An inclusive-fitness analysis. Journal of Evolutionary Biology 33:1806-1812.

Hamilton, W. D. 1972. Altruism and related phenomena, mainly in social insects. Annu. Rev. Ecol. Syst. 3:193-232.

Hamilton, W. D. 1979. Wingless and fighting males in fig wasps and other insects. Pp. 167-220 *in* M. S. Blum, and N. A. Blum, eds. Sexual selection and reproductive competition in insects. Academic Press, Athens, Georgia.

Lehtonen, J. 2018. The Price equation, gradient dynamics, and continuous trait game theory. The American Naturalist 191:146-153.

Otto, S. P. and T. Day. 2007. A biologist's guide to mathematical modeling in ecology and evolution. Princeton University Press, Princeton.

Taylor, P. D. 1993. Female-biased sex ratios under local mate competition: An experimental confirmation. Evol. Ecol. 7:306-308.

Taylor, P. D. and S. A. Frank. 1996. How to make a kin selection model. Journal of Theoretical Biology 180:27-37.

West, S. A. 2009. Sex Allocation. Princeton University Press, Princeton.
